# Guignardones P–S, New Meroterpenoids from the Endophytic Fungus *Guignardia mangiferae* A348 Derived from the Medicinal Plant *Smilax glabra*

**DOI:** 10.3390/molecules201219890

**Published:** 2015-12-21

**Authors:** Zhang-Hua Sun, Fa-Liang Liang, Wen Wu, Yu-Chan Chen, Qing-Ling Pan, Hao-Hua Li, Wei Ye, Hong-Xin Liu, Sai-Ni Li, Guo-Hui Tan, Wei-Min Zhang

**Affiliations:** 1 State Key Laboratory of Applied Microbiology Southern China, Guangdong Provincial Key Laboratory of Microbial Culture Collection and Application, Guangdong Open Laboratory of Applied Microbiology, Guangdong Institute of Microbiology, Guangzhou 510070, China; sysuszh@126.com (Z.-H.S.); 15627860105@163.com (F.-L.L.); yuchan2006@126.com (Y.-C.C.); hutu70@163.com (Q.-L.P.); hhli100@126.com (H.-H.L.); yewei718@163.com (W.Y.); hxinliu1225@163.com (H.-X.L.); maibao66@126.com (S.-N.L.); 13430272007@163.com (G.-H.T.); 2 School of Chinese Materia Medica, Guangzhou University of Chinese Medicine, Guangzhou, Guangdong 510006, China; wuwen@gzucm.edu.cn

**Keywords:** *Guignardia mangiferae*, meroterpenoids, structure identification, *Smilax glabra*, endophytic fungus

## Abstract

Four new meroterpenoids, guignardones P–S (**1**–**4**), and three known analogues (**5**–**7**) were isolated from the endophytic fungal strain *Guignardia mangiferae* A348. Their structures were elucidated on the basis of spectroscopic analysis and single crystal X-ray diffraction. All the isolated compounds were evaluated for their inhibitory effects on SF-268, MCF-7, and NCI-H460 human cancer cell lines. Compounds **2** and **4** exhibited weak inhibitions of cell proliferation against MCF-7 cell line.

## 1. Introduction

**Figure 1:**
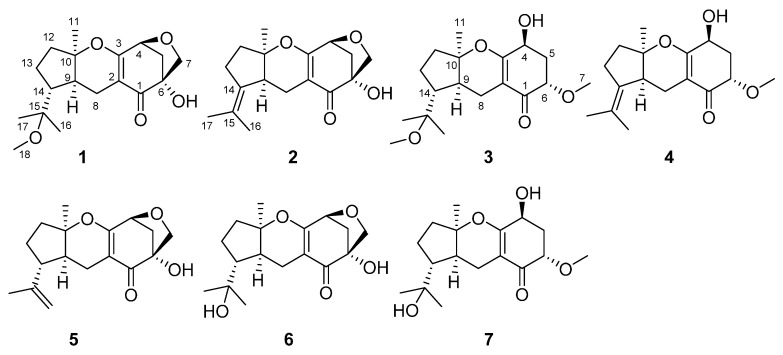
The chemical structures of compounds **1**–**7**.

Endophytic fungi that reside in plants are promising sources of a variety of bioactive metabolites. These metabolites are usually structurally novel and display important biological or pharmaceutical properties, such as antimicrobial or cytotoxic activities [1,2,3]. *Smilax glabra* is a common wild plant and has been used in folk medicine for the treatment of brucellosis, syphilis, acute and chronic nephritis, and metal poisoning [4,5,6,7]. In this study, the endophytic fungal strain *Guignardia mangiferae* A348 was isolated from leaves of *S. glabra* collected in Luofu Mountain Natural Reservation of China. Previous chemical investigations of the genus *guignardia* yielded several bioactive secondary metabolites, including meroterpenoids, spirodioxynaphthalenes, vermistatin and penicillide derivatives [8,9,10,11,12]. As part of an ongoing program aimed at exploring the secondary metabolites of fungi obtained from medicinal plants, we previously isolated several sterols, and aliphatics from the strain *G. mangiferae* A348 derived from *Smilax glabra* [13]. Continued chemical investigation of laboratory cultures of *G. mangiferae* A348 resulted in the isolation of seven meroterpenoids (Figure 1), including four new analogues, guignardones P–S (**1**–**4**). Compounds **1**–**7** were evaluated for their cytotoxicities against SF-268, MCF-7, and NCI-H460 cell lines. Herein, the isolation, structure elucidation, and the inhibitory activities of these meroterpenoids are described.

## 2. Results and Discussion

The fermentation broth of the endophytic fungal strain *G. mangiferae* A348 was extracted with EtOAc and then concentrated under reduced pressure to give an extract. The EtOAc extract was subjected to various column chromatography protocols to afford compounds **1**–**7**. These new structures were identified by spectroscopic analyses and physicochemical properties, while the known analogues were identified as guignardone A (**5**) [14], guignardone B (**6**) [14], and guignardone I (**7**) [15] by comparison of their spectroscopic data and specific rotations with those in the literature.

### 2.1. Identification of New Compounds

**Figure 2:**
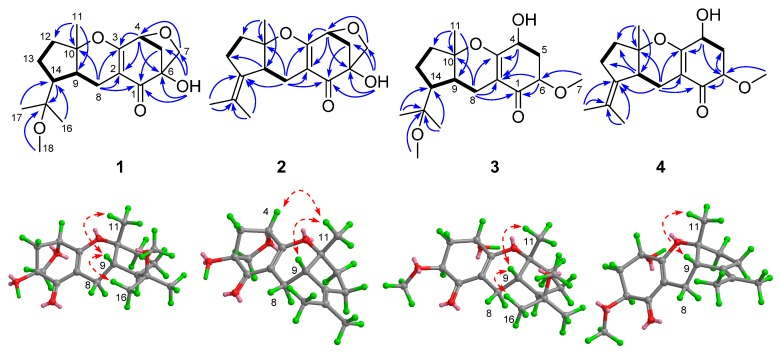
Key ^1^H-^1^H COSY (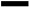), HMBC (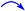), and NOE (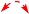) correlations of compounds **1**–**4**.

Compound **1**, a colorless crystal, had the molecular formula C_18_H_26_O_5_, as established by HREIMS, corresponding to six degrees of unsaturation. The ^1^H-NMR spectrum (Table 1) exhibited signals for four methyls [δ_H_ 3.08 (3H, s, H_3_-18, methoxy group), 1.29 (3H, s, H_3_-11), 1.09 (3H, s, H_3_-16), and 1.08 (3H, s, H_3_-17)], three oxymethine protons [δ_H_ 4.55 (1H, d, *J* = 5.4 Hz, H-4), 3.79 (1H, d, *J* = 7.9 Hz, H-7a), and 3.48 (1H, d, *J* = 7.9 Hz, H-7b)], and a series of aliphatic methylene multiplets. The ^13^C-NMR spectrum, in combination with HSQC experiment, resolved 18 carbon resonances attributable to a carbonyl (δ_C_ 198.6, C-1), a tetrasubstituted double bond [δ_C_ 173.7 (C-3) and 102.8 (C-2)], four methyls [δ_C_ 48.9 (C-18), 23.3 (C-17), 22.9 (C-11), and 21.9 (C-16)], five sp^3^ methylenes [δ_C_ 70.5 (C-7, bearing heteroatom), 43.9 (C-5), 38.0 (C-12), 24.4 (C-13), and 17.6 (C-8)], three sp^3^ methines [δ_C_ 78.4 (C-4, bearing heteroatom), 41.1 (C-9), and 48.3 (C-14)], and three sp^3^ quaternary carbons [81.6 (C-6), 90.7 (C-10), 76.7 (C-15)]). As two of the six degrees of unsaturation were accounted for by a carbonyl group, and a double bond, the remaining four degrees of unsaturation required that **1** was tetracyclic. The above mentioned information was similar to that of the known meroterpene, guignardone B (**6**), a metabolite co-isolated in the current study, except for the presence of a methoxy group in **1**. HMBC correlations from H_3_-17, H_3_-16 (Figure 2), and the methoxy group to a 4 ppm downfield-shifted carbon C-15 (δ_C_ 76.7) revealed that the methoxy group was located at C-15. 

**Figure 3:**
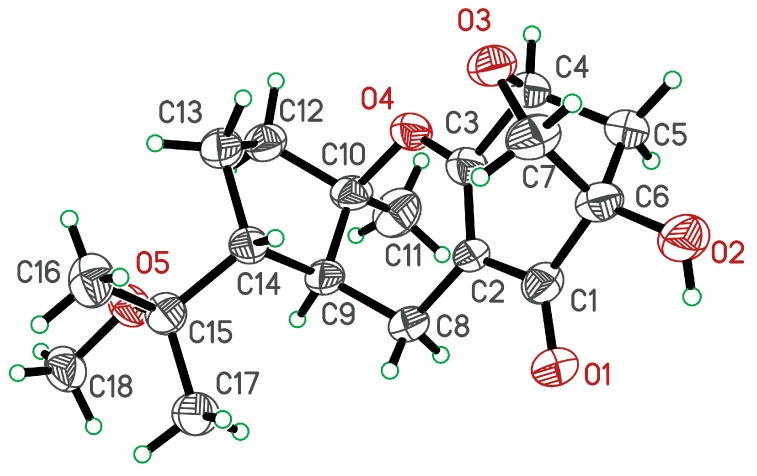
ORTEP diagram of compound **1**.

Detailed 2D analyses (HSQC, ^1^H-^1^H COSY, and HMBC) supported the planar structure of **1** as depicted. Compound **1** was further confirmed by its X-ray diffraction analysis (Figure 3), which also established its relative configuration. Thus, the structure of **1** was established and given the trivial name guignardone P.

**Table 1:** ^1^H-NMR data of **1**−**4** in CDCl_3_ at 500 MHz (*J* in Hz, δ in ppm).

Position	1	2	3	4
H-4	4.55, d (5.4)	4.58, d (5.4)	4.27, m	4.31, t (5.4)
H-5	2.45, dd (10.7, 5.5)	2.44, dd (10.7, 5.5)	2.41, m	2.43, m
	2.02, d (10.7)	2.04, d (10.7)	2.24, m	2.19, m
H-6			3.72, dd (7.5, 3.8)	3.73 dd (7.5, 3.8)
H-7	3.79, d (7.9)	3.80, d (7.9)	3.49, s	3.49, s
	3.48, d(7.9)	3.51, d (7.9)		
H-8	2.66, dd (17.0, 1.2)	2.63, dd (17.2, 7.2)	2.66, d (17.2)	2.64, dd (17.2, 7.2)
	2.20, dd (17.0, 6.1)	1.88, m	2.20, m	1.85, m
H-9	2.04, m	2.39, t (8.3)	2.04, m	2.47, m
H-11	1.29, s	1.35, s	1.33, s	1.39, s
H-12	1.98, m	1.85, m	2.01, m	1.89, m
	1.62, m	1.81, m	1.60, m	1.80, m
H-13	1.72, m	2.34, m	1.72, m	2.32, m
	1.52, m	2.21, m	1.60, m	2.24, m
H-14	1.71, m		1.74, m	
H-16	1.09, s	1.69, s	1.12, s	1.71, s
H-17	1.08, s	1.57, s	1.12, s	1.59, s
H-18	3.08, s		3.12, s	
OH	4.26, brs	4.24, brs	3.24, d (7.2)	

Compound **2** displayed a molecular ion at *m*/*z* 313.1392 [M + Na]^+^, consistent with a molecular formula of C_17_H_22_O_4_ as established by HRESIMS, 32 mass units less than that of **1**. The ^1^H- and ^13^C-NMR data of **2** (Table 1 and Table 2) were very similar to those of **5**, implying that **2** was a tricycloalternarene. The structural differences between **2** and **5** were attributed to the different locations of the double bonds at C-14, as the HMBC correlations from H_3_-16 and H_3_-17 to C-14 in **2** revealed that the double bond was located at C-14 and C-15. Detailed 2D analyses (HSQC, ^1^H-^1^H COSY, and HMBC) revealed the planar structure of **2** as depicted (Figure 3). The relative configuration of **2** was assigned to be the same as that of **1** by comparing their 1D NMR data and by analyzing its NOESY data. In particular, the NOESY correlations of H-9/H_3_-11, and H_3_-11/H-4 indicated that H-4, H-9, H_3_-11 and OH-6 were co-facial and arbitrarily assigned in α-oriented. Thus, the structure of **2** was established as depicted in Figure 1 and was given the trivial name guignardone Q.

**Table 2:** ^13^C-NMR data of **1**−**4** in CDCl_3_ at 125 MHz (*J* in Hz, δ in ppm).

Position	1	2	3	4
1	198.6	198.1	194.9	194.5
2	102.8	104.5	105.7	107.2
3	173.7	172.2	168.3	168.0
4	78.4	78.2	65.8	66.0
5	43.9	44.0	34.6	34.7
6	81.6	81.6	79.1	78.9
7	70.5	70.4	58.4	58.3
8	17.6	19.6	18.3	19.8
9	41.1	41.4	41.1	41.9
10	90.7	86.5	89.0	85.7
11	22.9	25.4	22.3	25.3
12	38.0	34.7	38.2	36.4
13	24.4	25.2	24.4	25.3
14	48.3	133.8	49.2	134.0
15	76.7	125.1	76.8	125.2
16	21.9	20.3	22.0	20.5
17	23.3	20.9	23.0	21.0
18	48.9		49.0	

Compound **3** was obtained as a white powder with a molecular formula C_18_H_28_O_5_ as established by HRESIMS at *m*/*z* 347.1814 [M + Na]^+^ (calcd 347.1834). The ^1^H-NMR spectrum of **3** displayed signals for five methyls (including two methoxy groups), two oxymethine protons, and a series of aliphatic methylene multiplets. The ^13^C-NMR spectrum, in combination with HSQC experiment, resolved 18 carbon resonances attributable to a carbonyl, a tetrasubstituted double bond, five methyls (including two methoxy group at δ_C_ 49.0 and 58.4), four sp^3^ methylene, four sp^3^ methines (two bearing heteroatom), and two sp^3^ quaternary carbons bearing oxygen atom. The aforementioned data were very similar to those of the co-isolated known meroterpene, guignardone I (**7**), except for the presence of an extra methoxyl at C-15 in **3**. Detailed 2D NMR analyses of **3** located the methoxyl at C-15 [HMBC correlation from H3-18 (δ_H_ 3.12) to C-15 (δ_C_ 76.8)]. The relative configuration of **3** was determined to be the same as **7** based on comparison of their ^1^H-^1^H coupling constants and chemical shifts. Thus, compound **3** was given the trivial name guignardone R.

HRESI(+)MS analysis of **4** revealed a highest mass *m*/*z* ion cluster consistent with a molecular formula (C_17_H_24_O_4_), requiring six double bond equivalents (DBE). Comparison of the NMR spectroscopic data for **4** with those for **3** revealed common subunits C-1 to C-8 and C-10 to C-12 accounting for five DBE, with the significant differences attributed to the presence of the non-conjugated double bond (δ_C_ 134.0 and 125.2; C-14 and C-15) in **4** instead of the methine (C-14) and oxygenated quaternary carbon (δ_C_ 76.8) in **3**. The gross structure of **4** was fully determined by the HMBC spectrum (Figure 3) and the stereochemistry was determined to be the same as that of **3** on the basis of analysis of its ^1^H-^1^H coupling constant and NOESY data. Thus, compound **4** was deduced as depicted and named guignardone S.

### 2.2. Cytotoxicity Assay

The *in vitro* cytotoxicities of compounds **1**–**7** were evaluated against three cancer cell lines, including SF268, MCF7, and NCI-H460. Compounds **2** and **4** exhibited weak growth inhibitions of cell proliferation against the cancer cell line MCF-7 with IC_50_ values of 83.7 and 92.1 μM, respectively.

## 3. Materials and Methods

### 3.1. General Experimental Procedures 

NMR spectra were recorded on a Bruker AVANCE 500 spectrometer (Bruker Corporation, Fremont, CA, USA) and referenced to the signals of tetramethylsilane as an internal standard. HREIMS was performed with an API QSTAR time-of-flight spectrometer (Thermo Fisher Scientific, Bremen, Germany) and HR-ESITOFMS were recorded on a Waters Acquity UPLC-Q-TOF Micro focus spectrometer (Waters Corp., Milford, MA, USA). X-ray structure determination: Rigaku R-AXIS SPIDER (Rigaku Corporation, Tokyo, Japan). UV spectra were recorded on a Biochrom Ultrospec 6300 pro UV-Visible spectrophotometer (GE Healthcare, London, UK). IR spectra were measured on a Perkin-Elmer Spectrum 100. A Shimadzu LC-20 AT (Shimadzu Corporation, Kyoto, Japan) equipped with an SPD-M20A PDA detector (Shimadzu Corporation) was used for HPLC, a YMC-pack ODS-A column (250 × 10 mm, 5 µm, 12 nm) was used for semipreparative HPLC separation and a YMC-pack ODS-A column (250 × 20 mm, 5 µm, 12 nm) was used for preparative HPLC separation. Column chromatography (CC, 250 × 40 mm): commercial silica gel (SiO_2_; 200–300 mesh; Qingdao Marine Chemical Plant, Qingdao, China). All solvents used were of analytical grade (Guangzhou Chemical Reagents Company, Ltd., Guangzhou, Guangdong, China).

### 3.2. Fungal Material

The endophytic fungal strain A348 was isolated from *Smilax glabra*, which was collected in Luofu Mountain Natural Reservation, Guangdong Province, China, in 17 November 2008. The isolated strain was identified as *Guignardia mangiferae* based on a morphological study and sequence analysis of rDNA ITS (internal transcribed spacer) with 99.8% similarity to the strain of *Guignardia mangiferae* ymy-11 (Accession No. EU677819) [13]. The strain is preserved at the State Key Laboratory of Applied Microbiology Southern China, Guangdong Institute of Microbiology.

### 3.3. Extraction and Isolation

The endophytic strain of *G. mangiferae* A348 was cultured in potato dextrose (PD) liquid medium, consisting of potato starch 20%, dextrose 2%, KH_2_PO_4_ 0.3%, MgSO_4_ 0.15%, Vitamin B1 10 mg/L. The cultivation was carried out at 28 °C with an agitation speed of 130 r/m for 7 days. The culture (100 L) was filtered to give the broth and mycelia. The broth was partitioned sequentially with EtOAc (3 × 300 mL) to yield a dark brown oily residue (13.8 g), which was subjected to column chromatography on silica gel using *n*-hexane as the first eluent and then acetone of increasing polarity to give six fractions (F1−F6). F3 (1397 mg) was purified on a preparative reversed-phase (RP) HPLC system equipped with a YMC column (MeOH/H_2_O, 50:50→100:0, 5 mL/min) to give nine subfractions (F3.1−F3.9). Subfraction F3.5 (43 mg) was purified on a semi-preparative reversed-phase RP-HPLC equipped with a YMC column (MeOH/H_2_O, 80:20, 3 mL/min) to give **7** (15.9 mg). Subfraction F3.6 (51 mg) was separated by RP-HPLC (YMC column, MeCN/H_2_O, 70:30, 3 mL/min) to yield **1** (6.2 mg), **3** (4.5 mg), and **6** (7.3 mg). Subfraction F3.7 (75 mg) was purified on a preparative RP-HPLC system equipped with a YMC column (MeCN/H_2_O, 80:20, 5 mL/min) to give **2** (3.3 mg), **4** (5.8 mg), and **5** (6.8 mg).

### 3.4. Spectroscopic Data

Guignardone P (**1**): colorless crystal (MeOH/H_2_O); m.p. 159–160 °C; ${\left[\alpha \right]}_{D}^{25}$ = 50 (*c* 0.1, MeOH); UV (MeOH) λ_max_ (log ε) 265 (3.41) nm; IR (KBr) ν_max_ = 3450, 2971, 2943, 2886, 1658, 1619, 1451, 1380, 1303, 1251, 1173, 1117, 1023 cm^−1^; HREIMS *m*/*z* 322.1778 (calcd for C_18_H_26_O_5_, 322.1780), composition for C_18_H_26_O_5_; ^1^H and ^13^C-NMR data, see Table 1 and Table 2.

*Guignardone* *Q* (**2**): white, amorphous powder; ${\left[\alpha \right]}_{D}^{25}$ = 39 (*c* 0.1, MeOH); UV (MeOH) λ_max_ (log ε) 265 (5.49) nm; IR (KBr) ν_max_ = 3494, 2928, 2856, 1741, 1653, 1617, 1460, 1382, 1281, 1248, 1077 cm^−1^; HRESIMS [M + Na]^+^ *m*/*z* 313.1392 (calcd for C_17_H_22_O_4_Na [M + Na]^+^, 313.1416); ^1^H- and ^13^C-NMR data, see Table 1 and Table 2.

*Guignardone* *R* (**3**): white, amorphous powder; ${\left[\alpha \right]}_{D}^{25}$ = −25 (*c* 0.1, MeOH); UV (MeOH) λ_max_ (log ε) 265 (2.79) nm; IR (KBr) ν_max_ = 3377, 2928, 2855, 1738, 1661, 1615, 1459, 1384, 1284, 1250, 1168, 1076, 1027 cm^−1^; HRESIMS [M + Na]^+^ *m*/*z* 347.1814 (calcd for C_18_H_28_O_5_Na [M + Na]^+^, 347.1834); ^1^H- and ^13^C-NMR data, see Table 1 and Table 2.

*Guignardone* *S* (**4**): colorless oil; ${\left[\alpha \right]}_{D}^{25}$ = −17 (*c* 0.1, MeOH); UV (MeOH) λ_max_ (log ε) 262 (2.87) nm; IR (KBr) ν_max_ = 3430, 2930, 2855, 1737, 1617, 1452, 1363, 1246, 1078, 1027 cm^−1^; HRESIMS [M + Na]^+^ *m*/*z* 315.1530 (calcd for C_17_H_24_O_4_Na [M + Na]^+^, 315.1572); ^1^H- and ^13^C-NMR data, see Table 1 and Table 2.

### 3.5. X-ray Crystallographic Data

X-ray crystallographic study of Guignardone P (**1**): C_36_H_51_O_10_, M = 643.77 g/mol, orthorhombic, 0.293 × 0.138 × 0.098 mm^3^, space group P2_1_ (no. 4), *a* = 11.709 (2) Å, *b* = 10.027(2) Å, *c* = 14.378(3) Å, *α* = γ = 90 °, β = 92.49(3) °, V = 1686.5(6) Å^3^, Z = 2, D_c_ = 1.268 g·cm^−3^, F(000) = 694.0, Xcalibur, Onyx, Nova, Mo K*α* radiation, λ = 0.71073 Å, T = 293 K, 5.98° ≤ 2θ ≤ 54.96°, 16,119 reflections collected, 7576 unique (R_int_ = 0.0662). Final GooF = 1.071, R_1_ = 0.0857, wR_2_ = 0.2459, R indices based on 7576 reflections with *I* > 2sigma(I) (refinement on *F*^2^), 424 parameters, 42 restraint. Lp and absorption corrections applied, m = 0.091 mm^−1^. Flack parameter = 0.00 (10). Crystallographic data for the structure of **1** have been deposited in the Cambridge Crystallographic Data Centre with the deposition number CCDC 1431464. These data can be obtained free of charge via http://www.ccdc.cam.ac.uk/conts/retrieving.html (or from the CCDC, 12 Union Road, Cambridge CB2 1EZ, UK; Fax: +44-1223-336033; E-Mail: deposit@ccdc.cam.ac.uk).

### 3.6. Cytotoxicity Assay

The cell growth inhibitory activities of compounds **1**–**7** against human cancer cell lines SF-268, MCF-7, and NCI-H460, were tested using the previously published methods [16].

## 4. Conclusions

There are increasing examples of tricycloalternarenes (TCAs) in the literature and most of them were isolated from the endophytic fungus derived from plant [15,17]. The genus *Guignardia* is a rich source of TCAs such as guignardones [18,19]. In our continuing investigation on the chemical constituents of endophytic fungus derived from the medicinal plant, four new meroterpenoids and three known analogues have been isolated from the endophytic fungus *Guignardia mangiferae* A348 derived from the medicinal plant *Smilax glabra*. The structures were determined by combined spectroscopic analysis and single crystal X-ray diffraction. All the isolates were evaluated for *in vitro* cytotoxicity against SF-268, MCF-7, and NCI-H460 cell lines, and both **2** and **4** exhibited weak inhibitory activities against MCF-7 cell line. Recently, guignardone B (**6**) and its analogues were reported to possess moderate inhibition of *Candida albicans* growth [12]. In this study, these new compounds not only enrich the chemical variety of meroterpenoids, but also may be important for the antifungal activities.

## Supplementary Materials

The ^1^H- and ^13^C-NMR data of **1**–**7**, HR-ESI-MS, 2D-NMR spectra of compounds **1**–**4** (Figures S1–S34) can be accessed at: http://www.mdpi.com/1420-3049/20/12/19890/s1.
