# Supplementary material for: Guignardones P–S, New Meroterpenoids from the Endophytic Fungus Guignardia mangiferae A348 Derived from the Medicinal Plant Smilax glabra
Source: Molecules. 2015 Dec 21;20(12):22900–7. doi: 10.3390/molecules201219890 (PMC6332344; doi:10.3390/molecules201219890)
Supplement: Supplementary file 1 [file molecules-20-19890-s001.pdf]

Supplementary Materials: Guignardones P–S, New Meroterpenoids from the Endophytic Fungi *Guignardia mangiferae* A348 Derived from the Medicinal Plant *Smilax glabra*

Zhang-Hua Sun, Fa-Liang Liang, Wen Wu, Yu-Chan Chen, Qing-Ling Pan, Hao-Hua Li, Wei Ye, Hong-Xin Liu, Sai-Ni Li, Guo-Hui Tan and Wei-Min Zhang

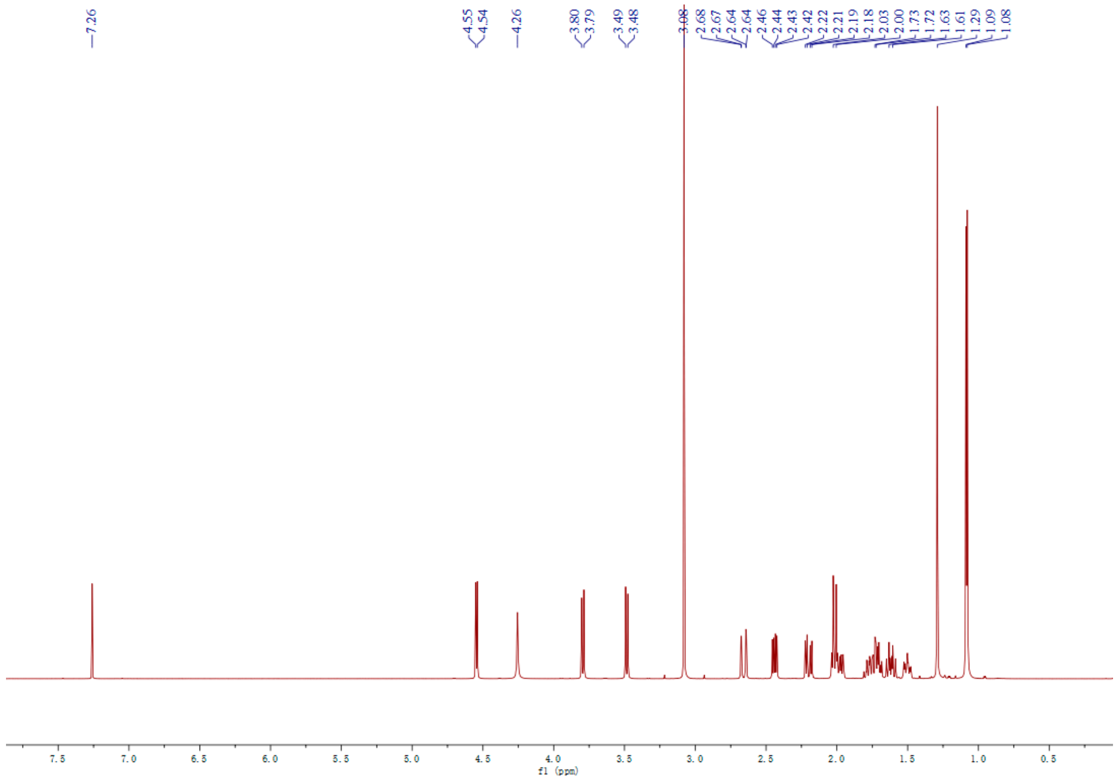

Figure S1. ^{1}H-NMR spectrum of guignardone P (**1**) in $CDCl_3$.

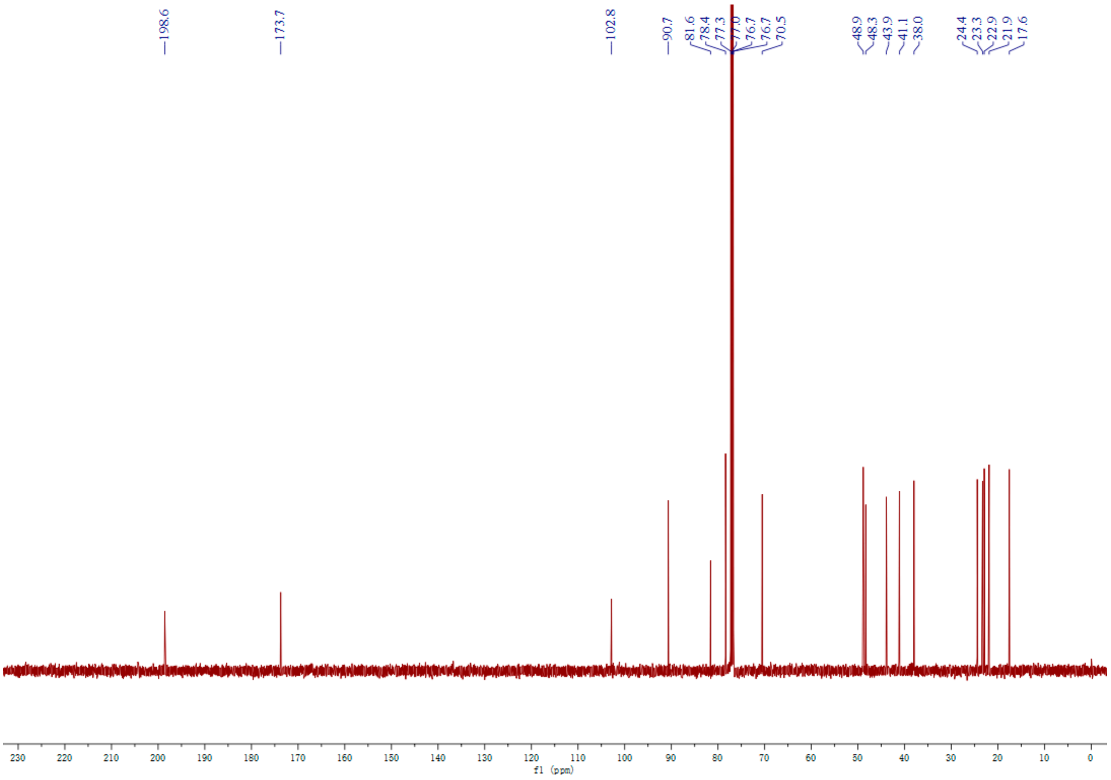

Figure S2. ^{13}C-NMR spectrum of guignardone P (**1**) in CDCl$_3$.

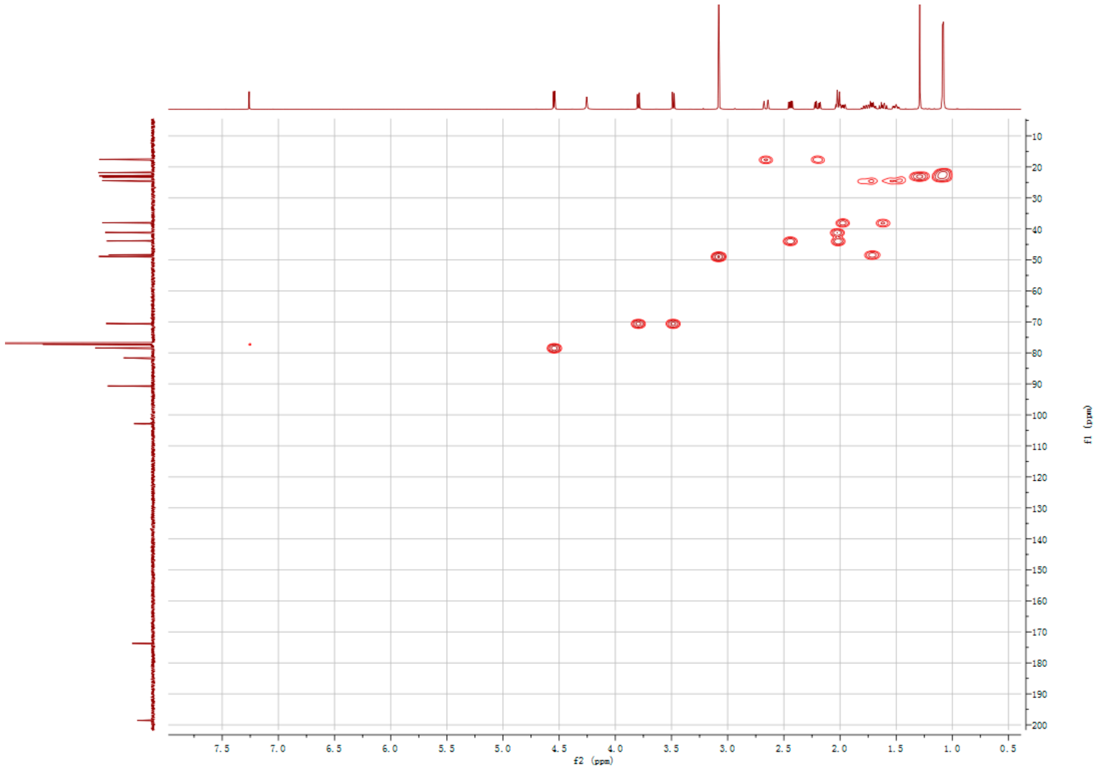

Figure S3. HSQC spectrum of guignardone P (**1**) in CDCl$_3$.

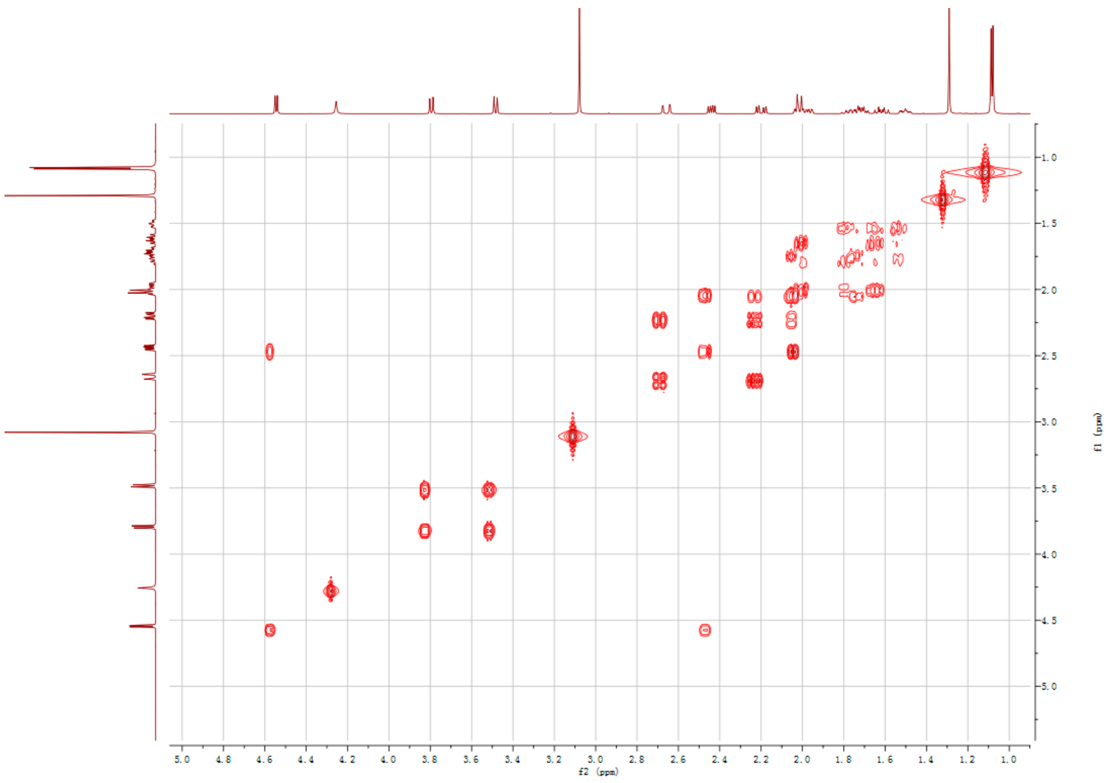

Figure S4. ^1H–^1H COSY spectrum of guignardone P (**1**) in $CDCl_3$.

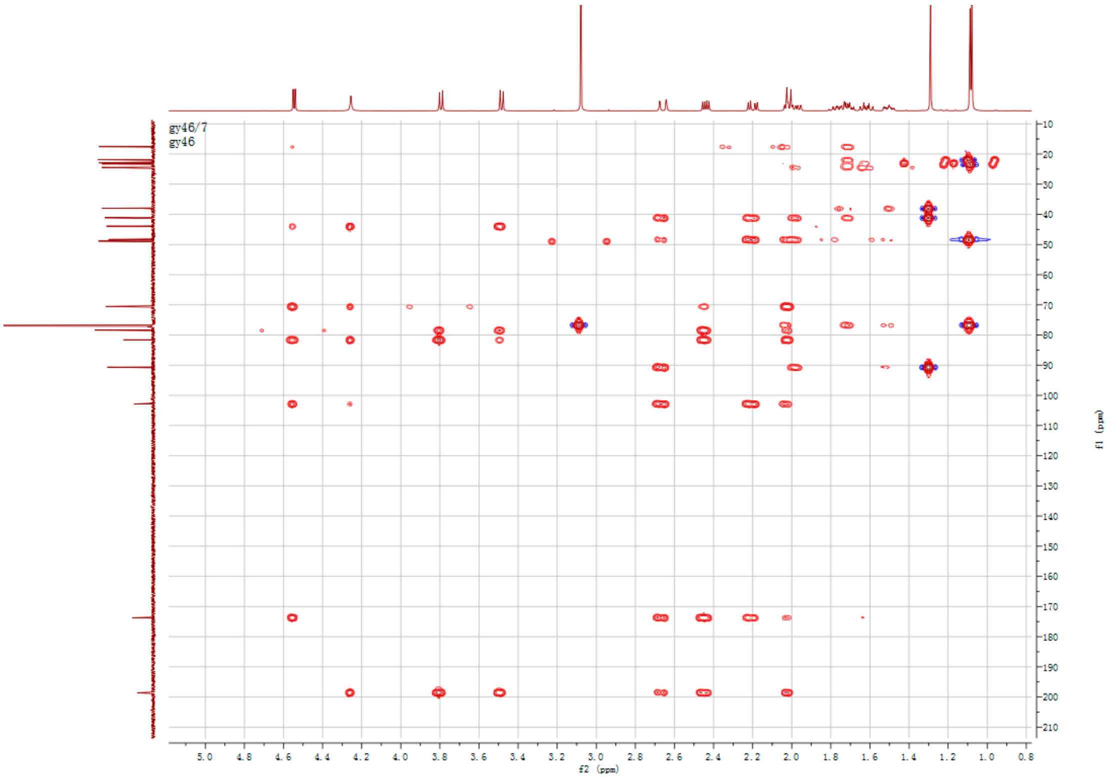

Figure S5. HMBC spectrum of guignardone P (**1**) in $CDCl_3$.

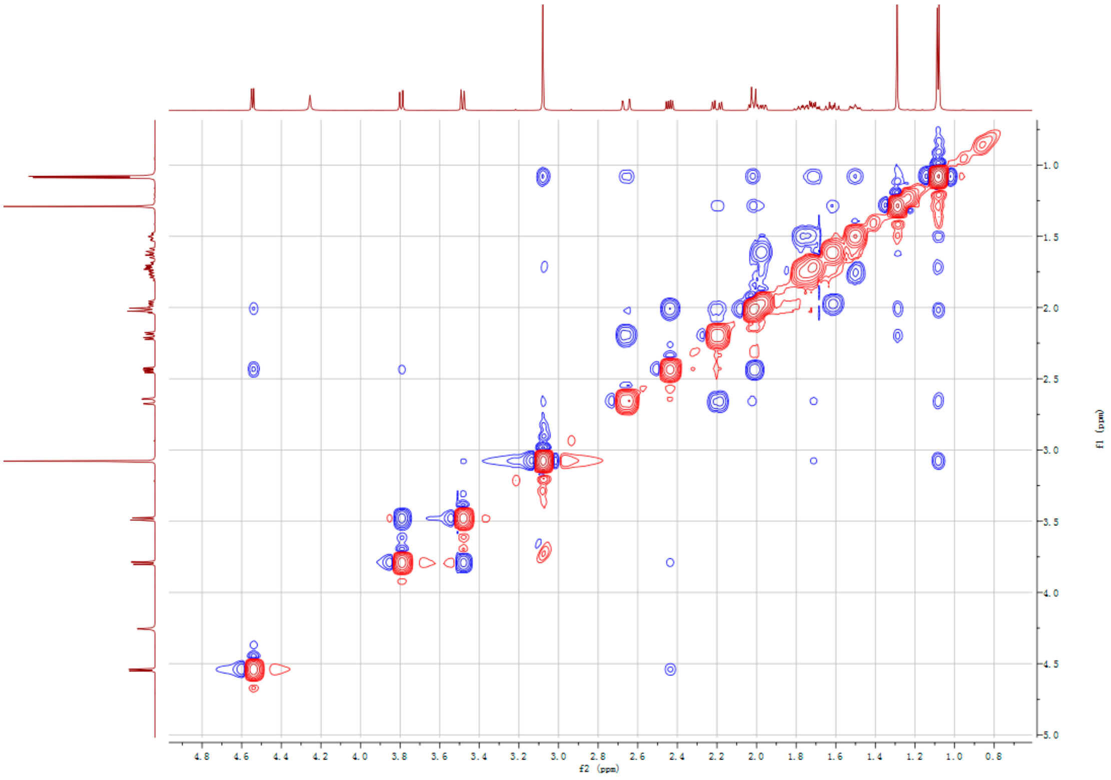

Figure S6. NOESY spectrum of guignardone P (**1**) in $CDCl_3$.

SPECTRUM - MS
File : D:\DATA-HR\11\051704-gy46-c1.RAW
Full ms [318.100 - 325.500] - Range: 318.100 - 325.500
Scan No. 30 of 39
Scan #: 30
RT: 0.51
Data points: 1

Mass	Relative Intensity	Theoretical Mass	Delta [ppm]	Delta [mmu]	RDB	Composition
322.1778	100.0	322.1775	0.9	0.3	6.0	C_{18} H_{26} O_5

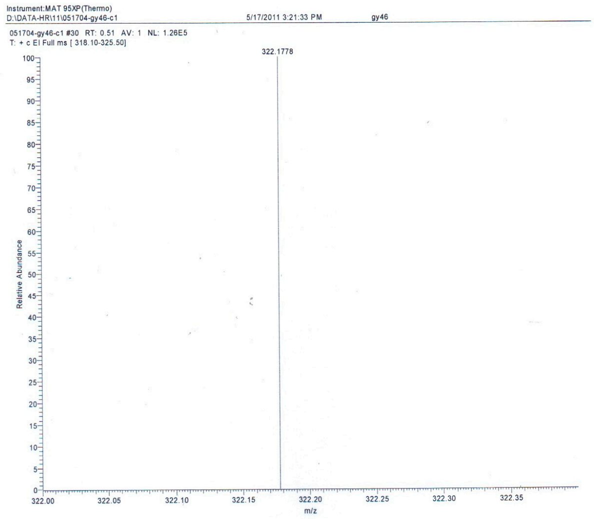

Figure S7. HREIMS spectrum of guignardone P (**1**).

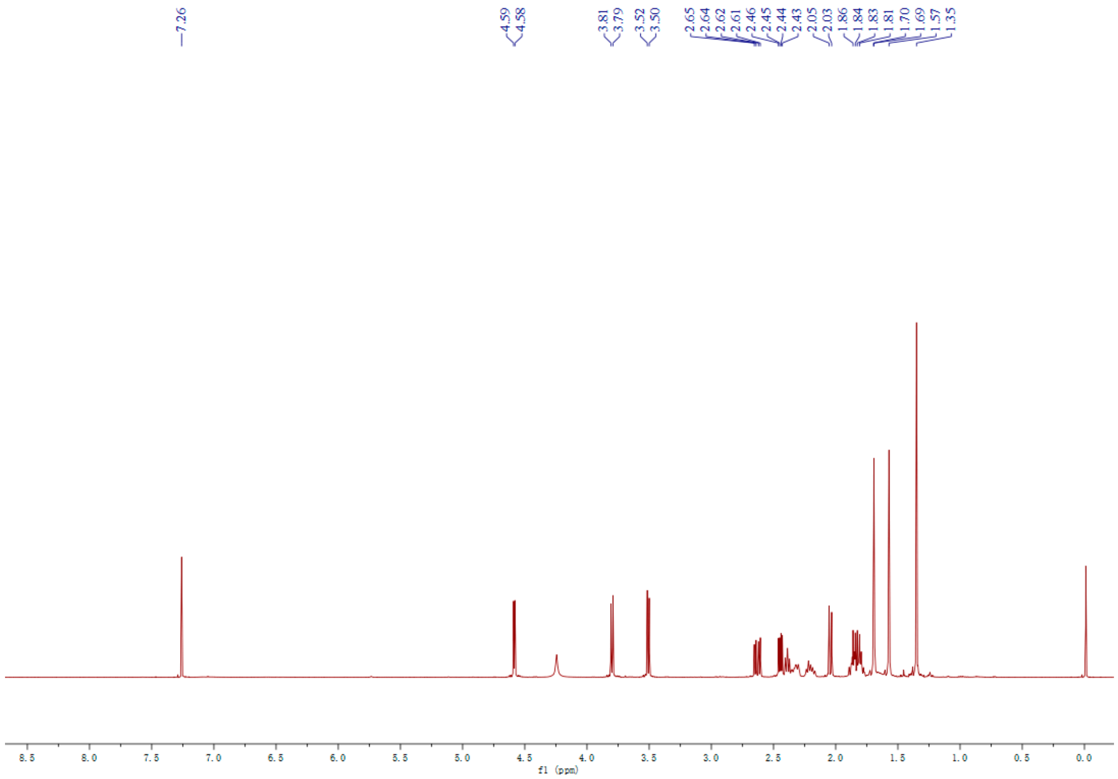

Figure S8. ^{1}H-NMR spectrum of guignardone Q (**2**) in $CDCl_3$.

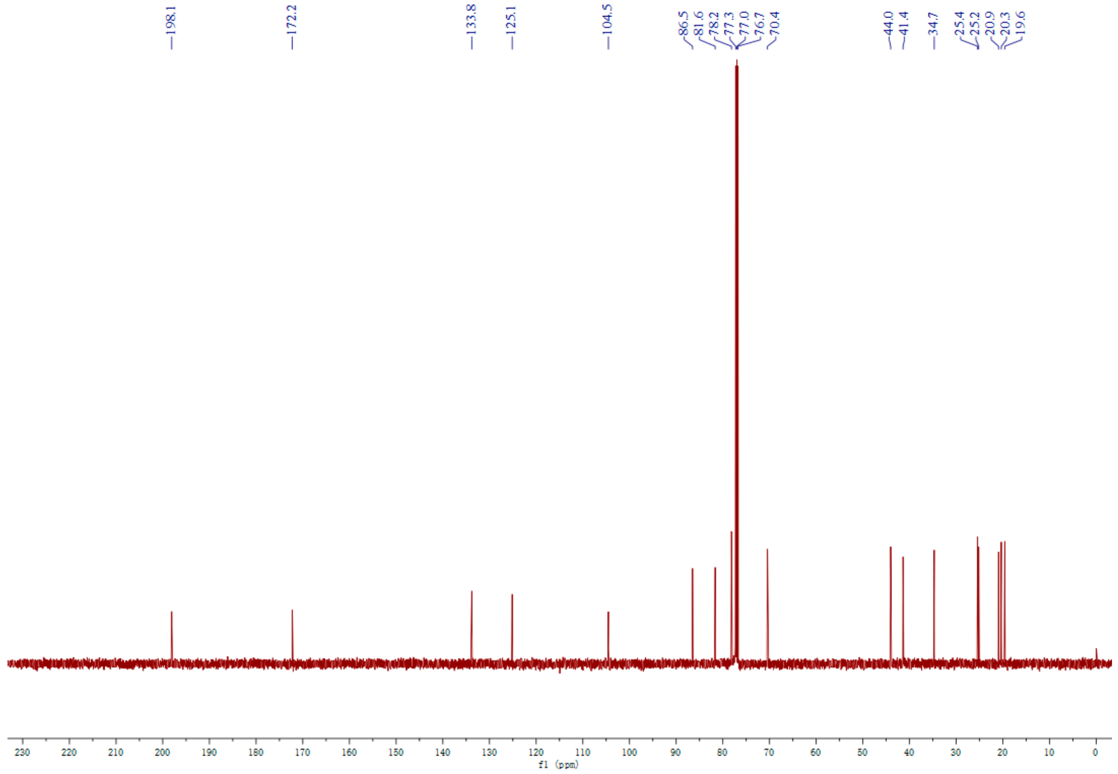

Figure S9. ^{13}C-NMR spectrum of guignardone Q (**2**) in $CDCl_3$.

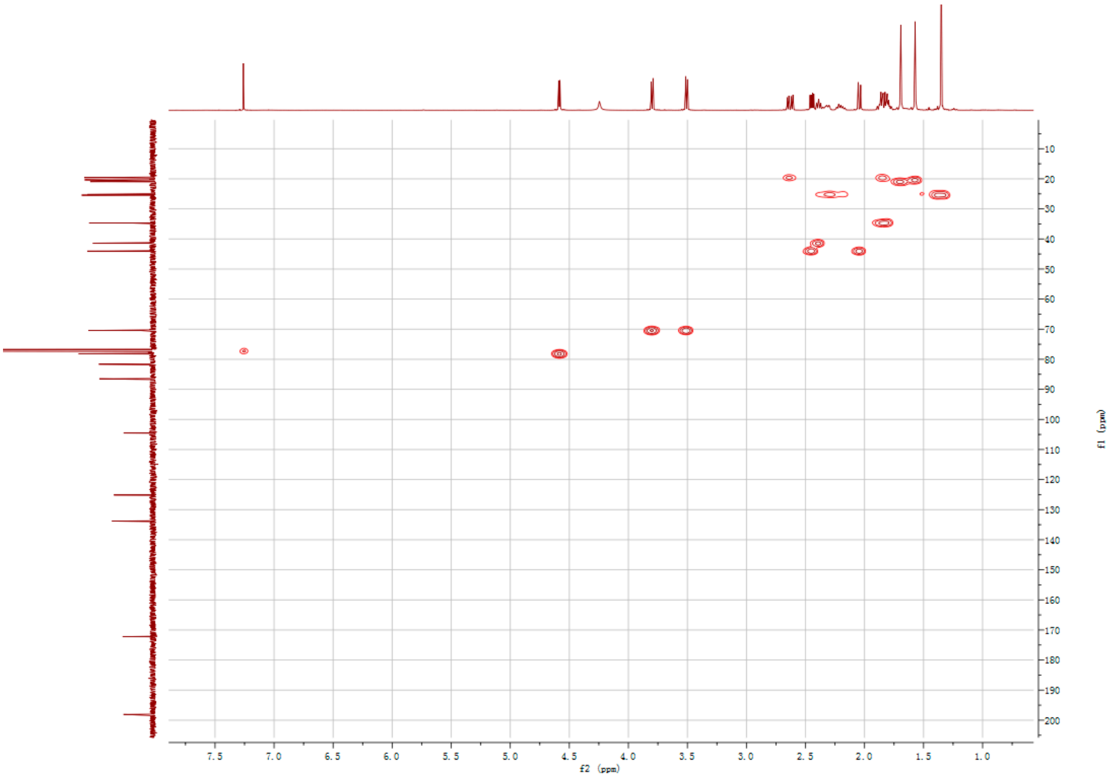

Figure S10. HSQC spectrum of guignardone Q (**2**) in $CDCl_3$.

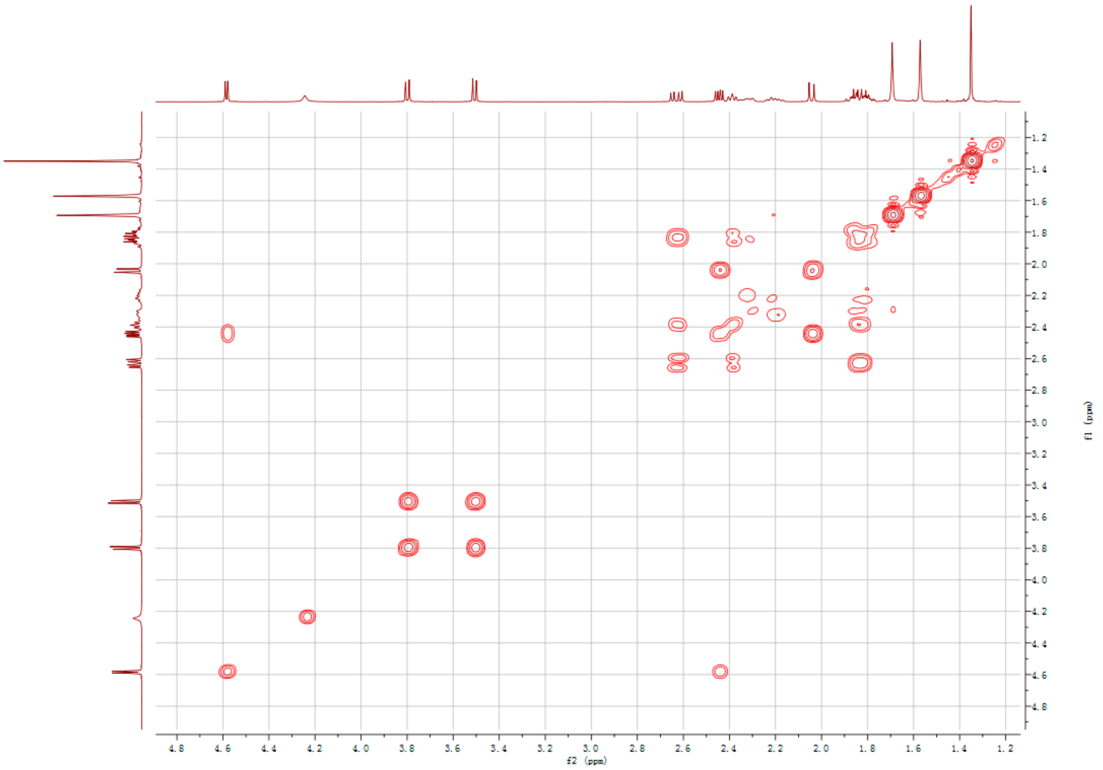

Figure S11. ^1H-^1H COSY spectrum of guignardone Q (**2**) in $CDCl_3$.

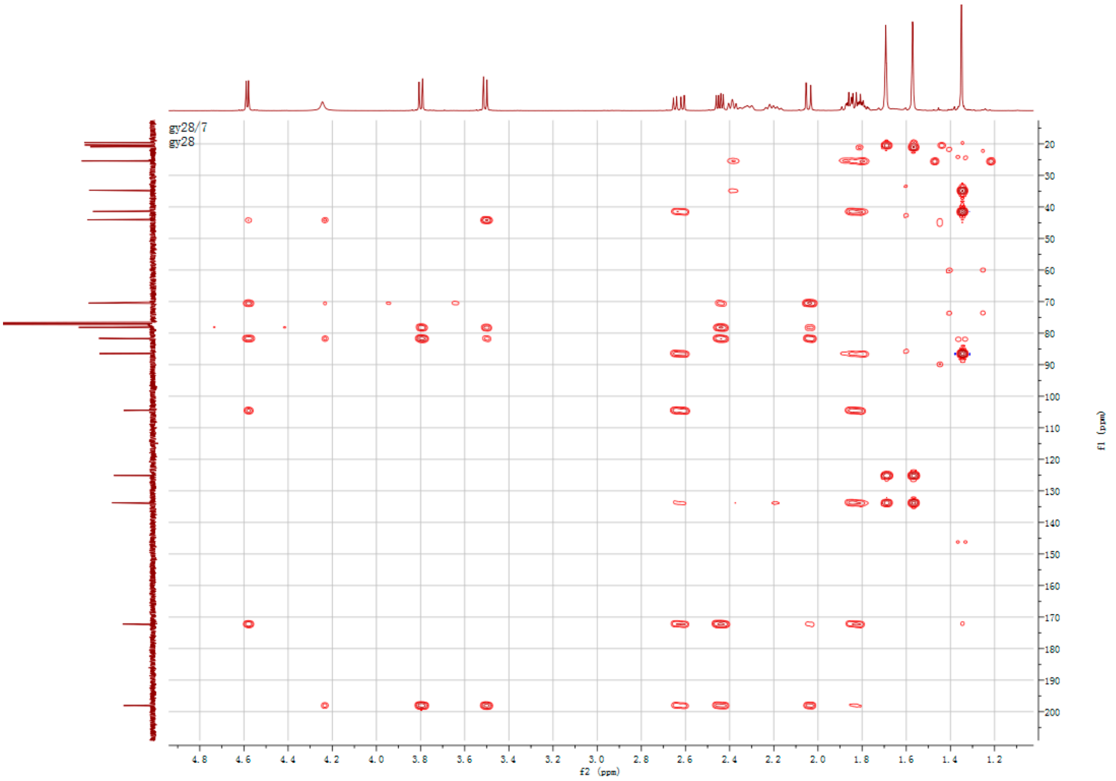

Figure S12. HMBC spectrum of guignardone Q (**2**) in $CDCl_3$.

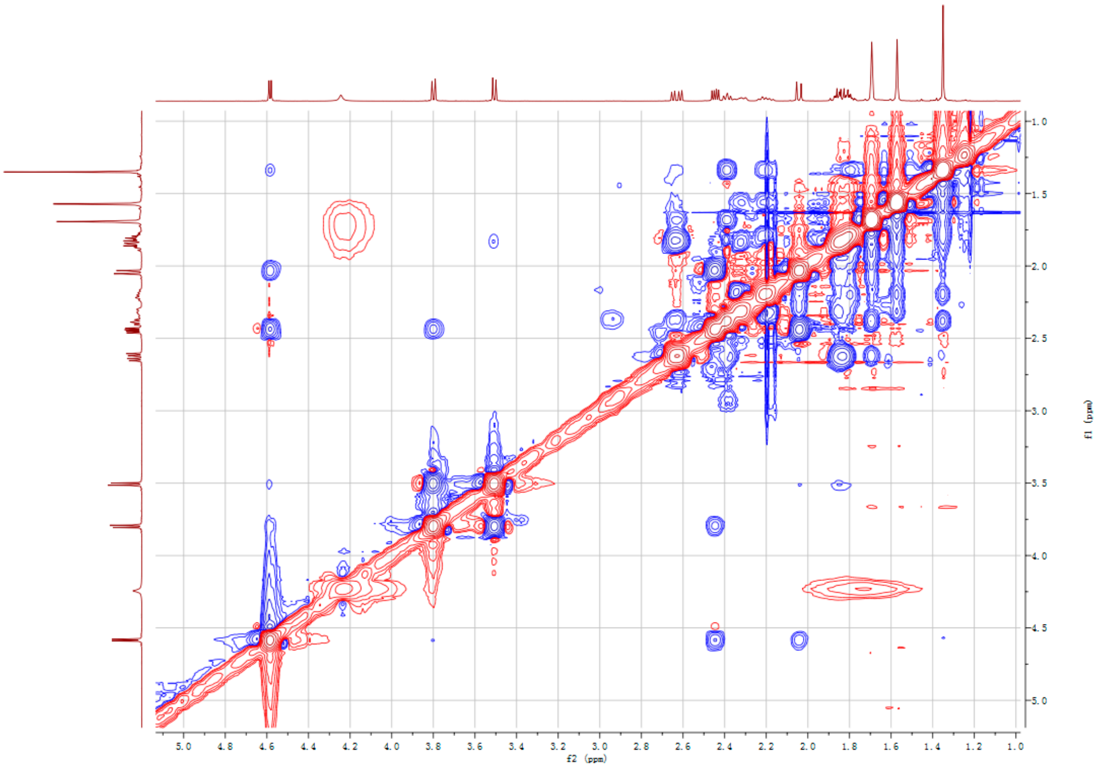

Figure S13. NOESY spectrum of guignardone Q (**2**) in $CDCl_3$.

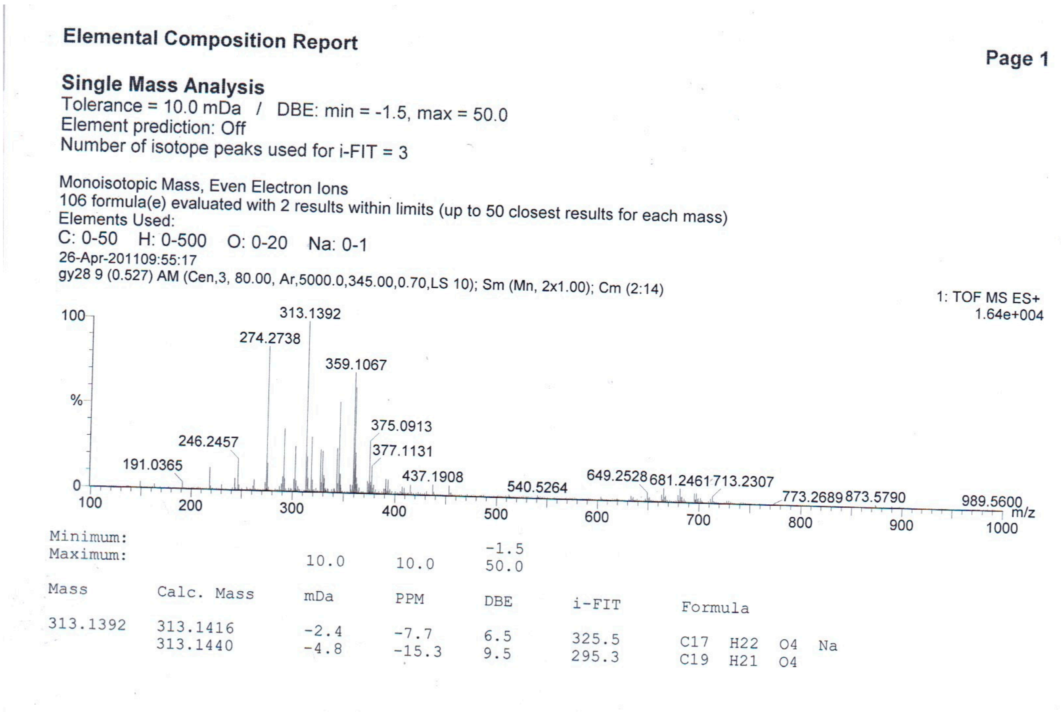

Elemental Composition Report Page 1

Single Mass Analysis
Tolerance = 10.0 mDa / DBE: min = -1.5, max = 50.0
Element prediction: Off
Number of isotope peaks used for i-FIT = 3

Monoisotopic Mass, Even Electron Ions
106 formula(e) evaluated with 2 results within limits (up to 50 closest results for each mass)
Elements Used:
C: 0-50 H: 0-500 O: 0-20 Na: 0-1
26-Apr-201109:55:17
gy28 9 (0.527) AM (Cen,3, 80.00, Ar,5000.0,345.00,0.70,LS 10); Sm (Mn, 2x1.00); Cm (2:14)

Minimum:				-1.5		
Maximum:		10.0	10.0	50.0		
Mass	Calc. Mass	mDa	PPM	DBE	i-FIT	Formula
313.1392	313.1416	-2.4	-7.7	6.5	325.5	C17 H22 O4 Na
	313.1440	-4.8	-15.3	9.5	295.3	C19 H21 O4

Figure S14. HRESIMS spectrum of guignardone Q (**2**).

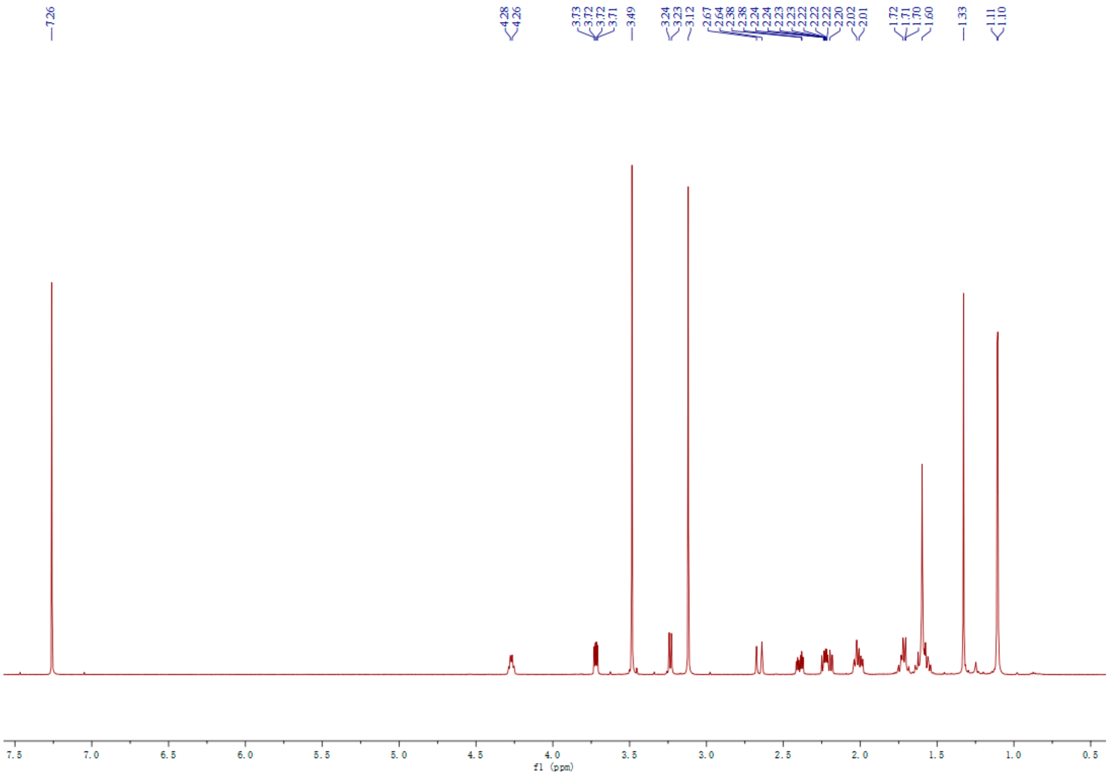

Figure S15. ^{1}H-NMR spectrum of guignardone R (**3**) in $CDCl_3$.

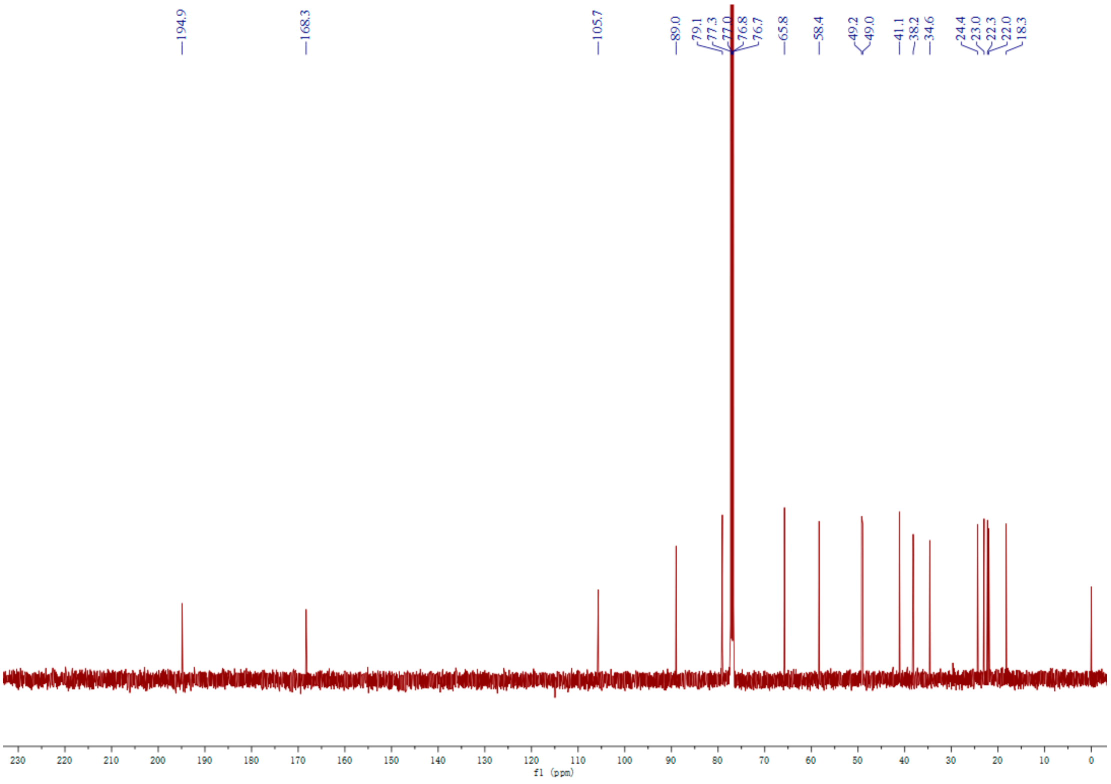

Figure S16. ^{13}C-NMR spectrum of guignardone R (**3**) in $CDCl_3$.

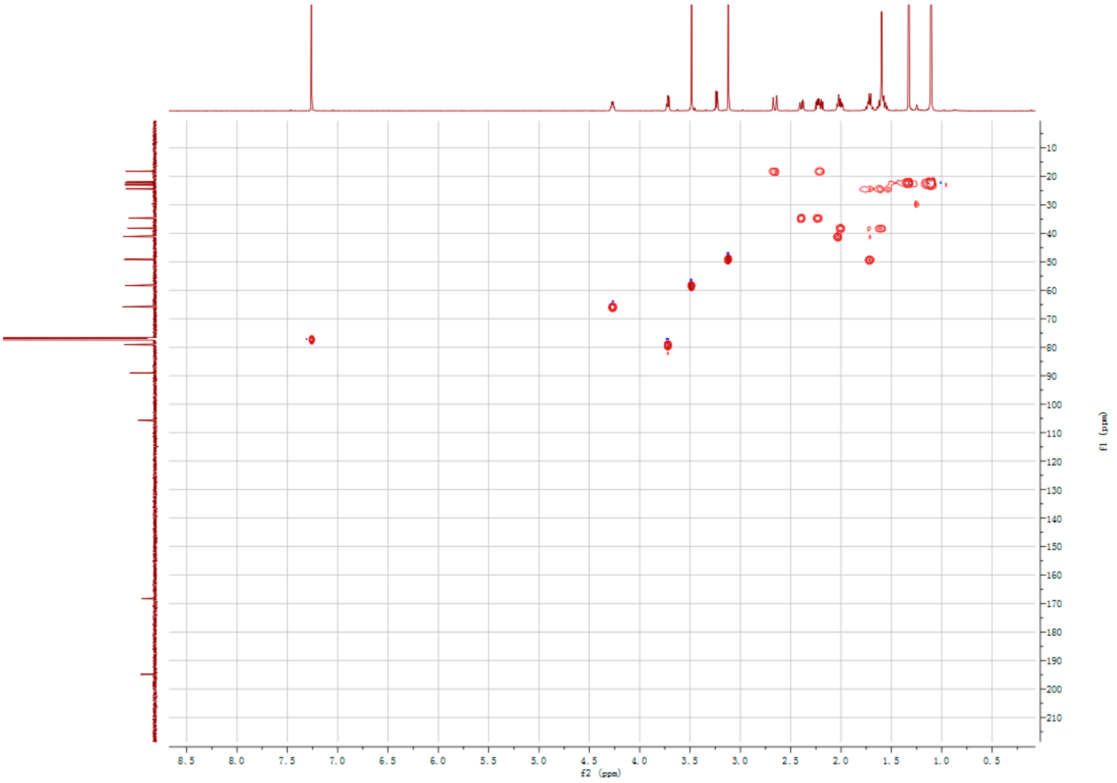

Figure S17. HSQC spectrum of guignardone R (**3**) in $CDCl_3$.

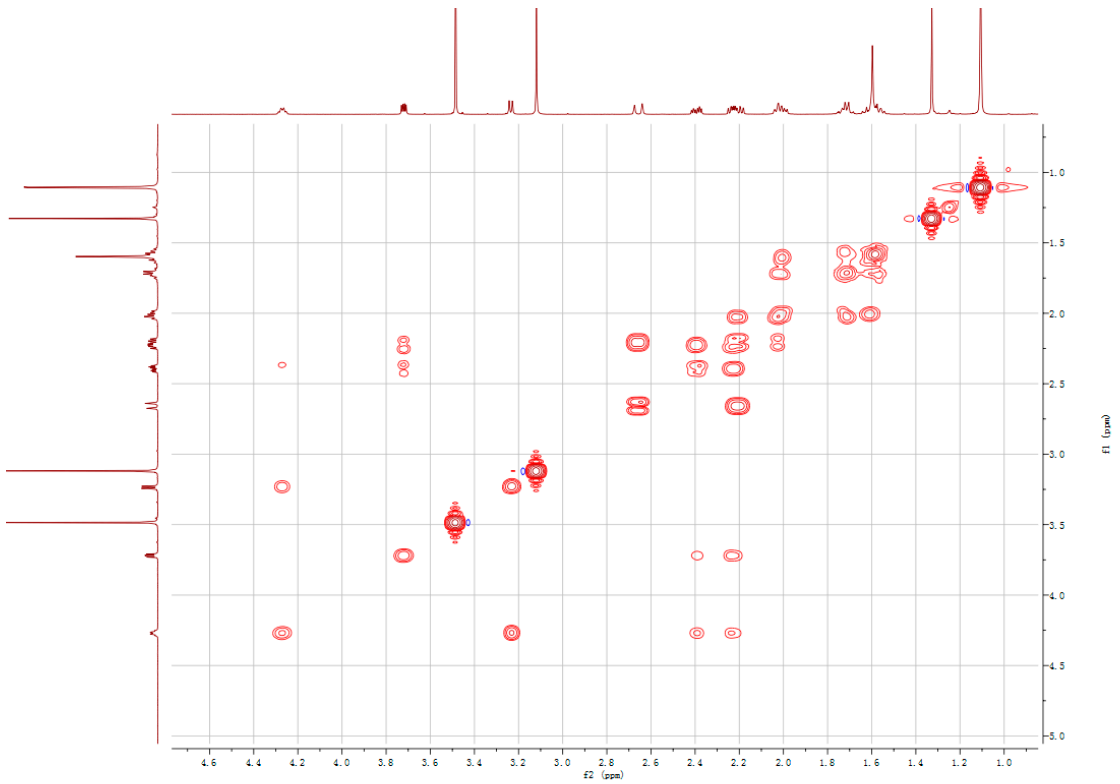

Figure S18. ^{1}H–^{1}H COSY spectrum of guignardone R (**3**) in $CDCl_3$.

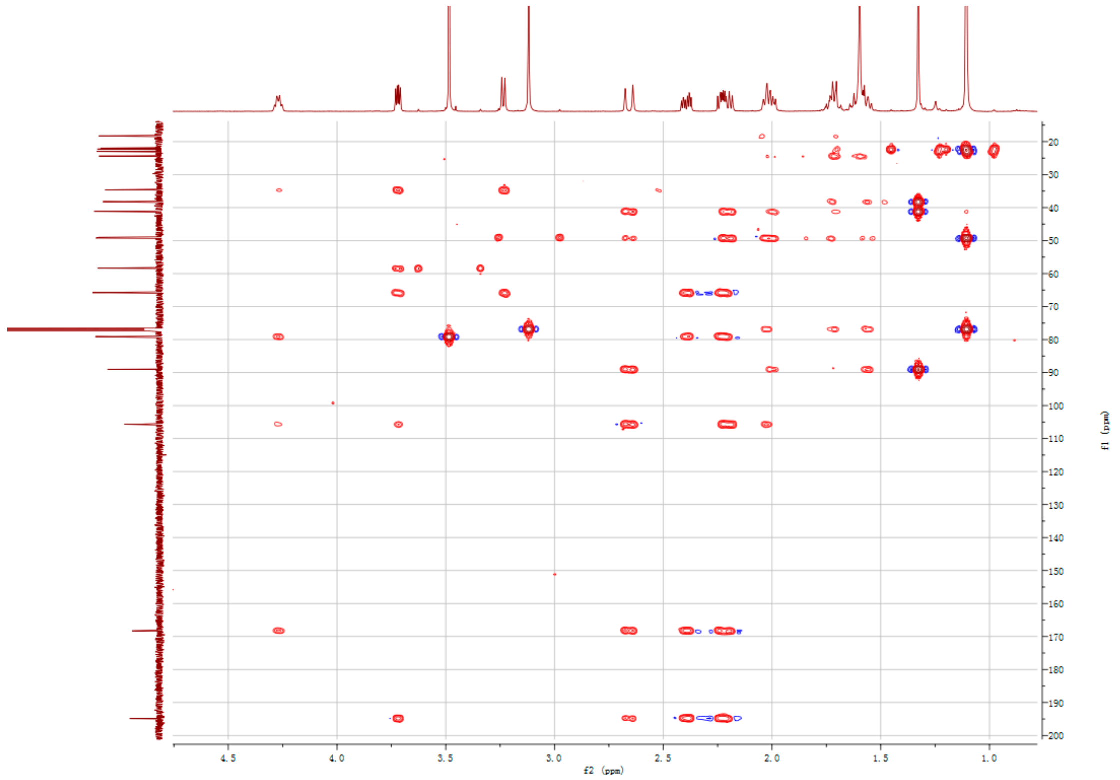

Figure S19. HMBC spectrum of guignardone R (**3**) in $CDCl_3$.

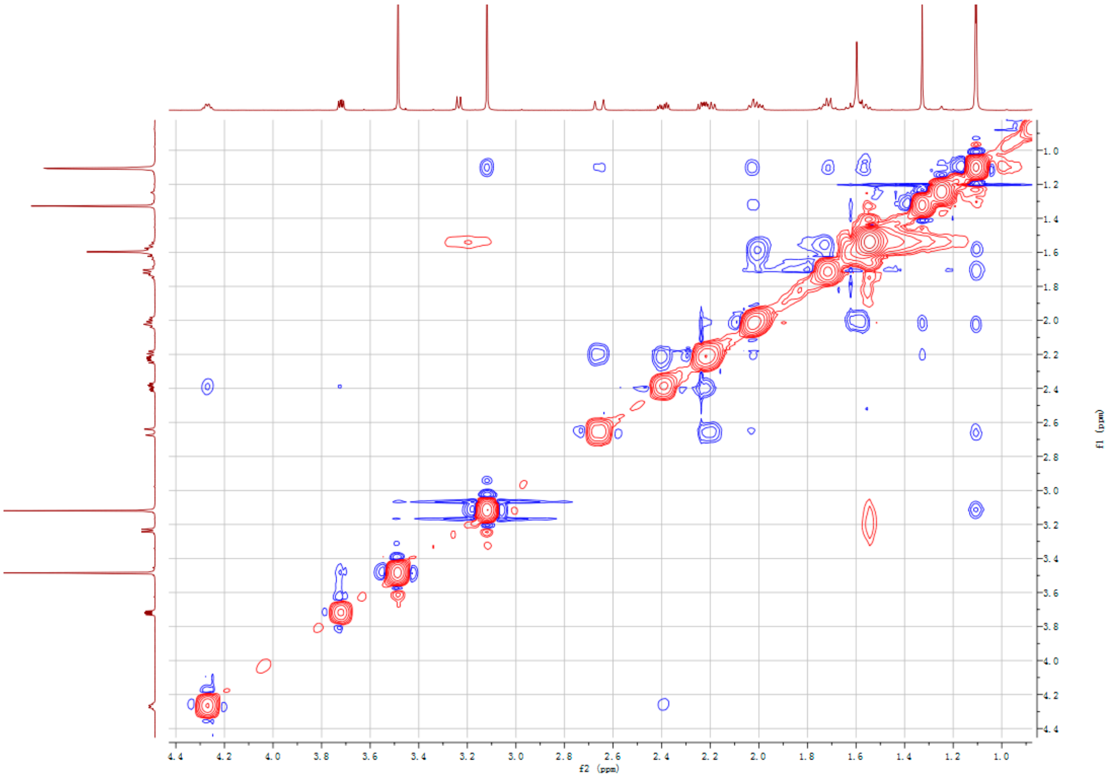

Figure S20. NOESY spectrum of guignardone R (**3**) in $CDCl_3$.

Elemental Composition Report **Page 1**

Single Mass Analysis
Tolerance = 10.0 mDa / DBE: min = -1.5, max = 50.0
Element prediction: Off
Number of isotope peaks used for i-FIT = 3

Monoisotopic Mass, Even Electron Ions
131 formula(e) evaluated with 4 results within limits (up to 50 closest results for each mass)
Elements Used:
C: 0-50 H: 0-500 O: 0-20 Na: 0-1
26-Apr-201110:10:23
gy37 5 (0.283) AM (Top,3, Ar,5000.0,345.00,0.70,LS 10); Sm (Mn, 2x1.00); Cm (4:20)

1: TOF MS ES+
1.49e+004

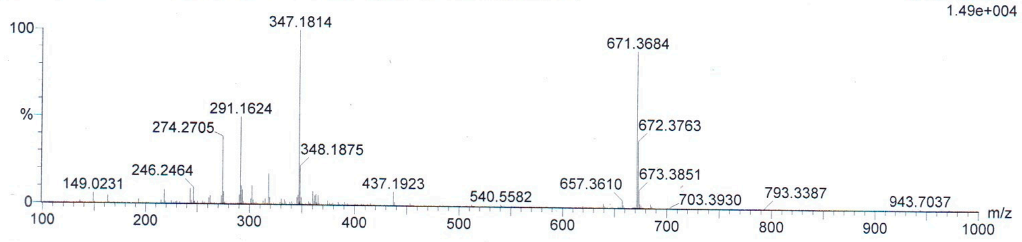

Minimum:				-1.5		
Maximum:		10.0	10.0	50.0		
Mass	Calc. Mass	mDa	PPM	DBE	i-FIT	Formula
347.1814	347.1800	1.4	4.0	16.5	168.4	C27 H23
	347.1834	-2.0	-5.8	4.5	28.8	C18 H28 O5 Na
	347.1776	3.8	10.9	13.5	107.3	C25 H24 Na
	347.1858	-4.4	-12.7	7.5	18.1	C20 H27 O5

Figure S21. HRESIMS spectrum of guignardone R (**3**).

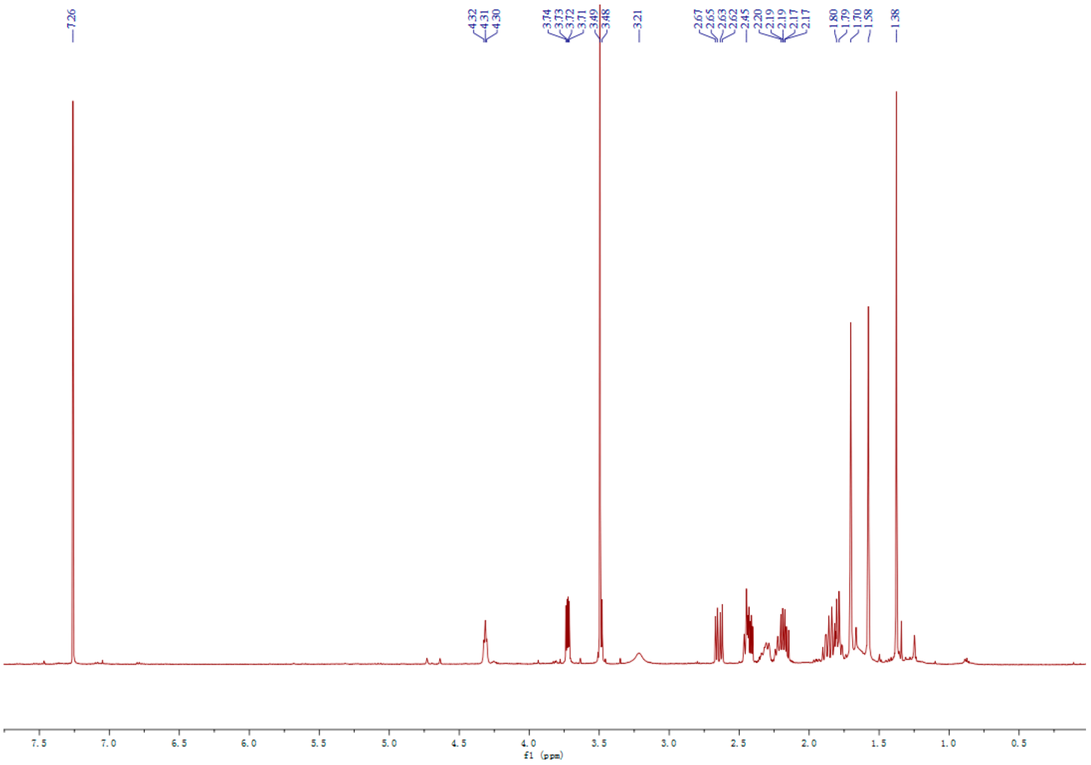

Figure S22. ^{1}H-NMR spectrum of guignardone S (**4**) in $CDCl_3$.

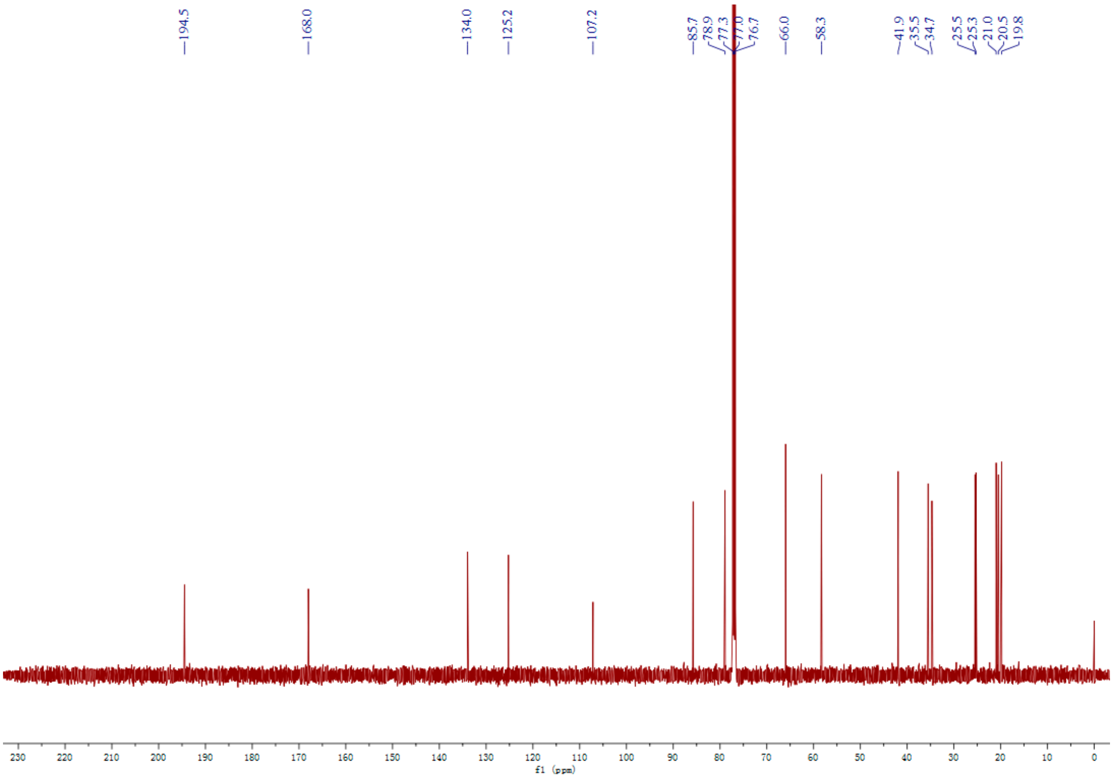

Figure S23. ^{13}C-NMR spectrum of guignardone S (**4**) in $CDCl_3$.

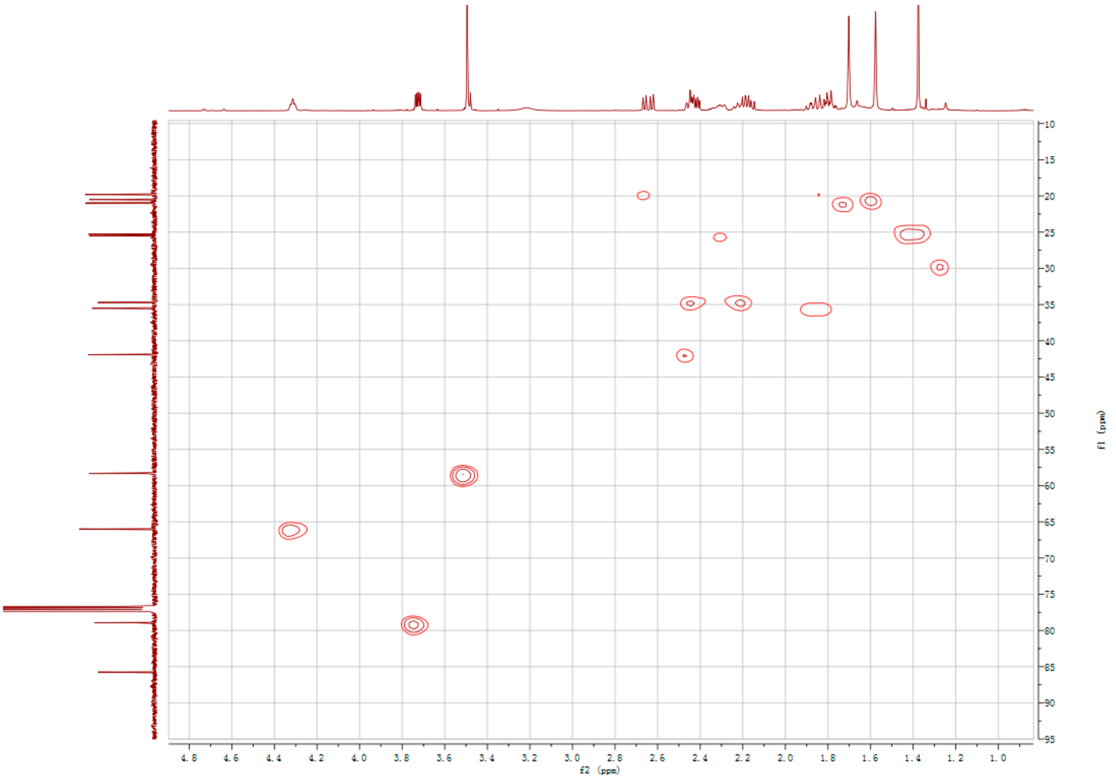

Figure S24. HSQC spectrum of guignardone S (**4**) in $CDCl_3$.

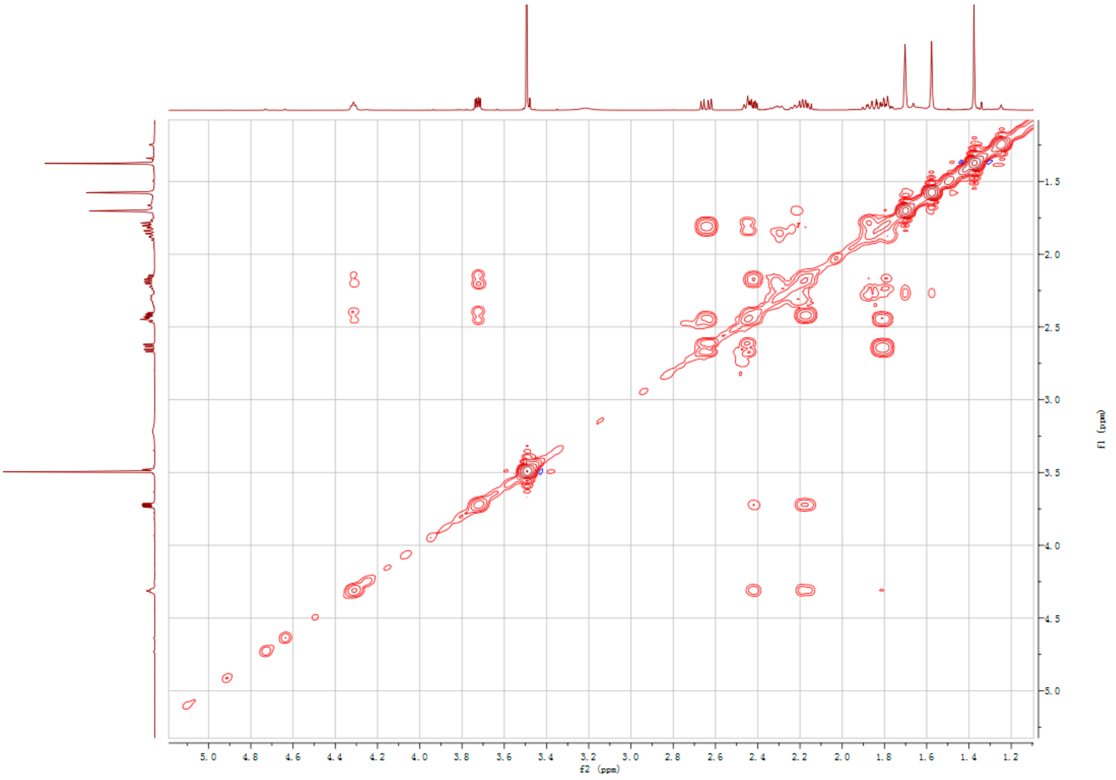

Figure S25. ^1H–^1H COSY spectrum of guignardone S (**4**) in $CDCl_3$.

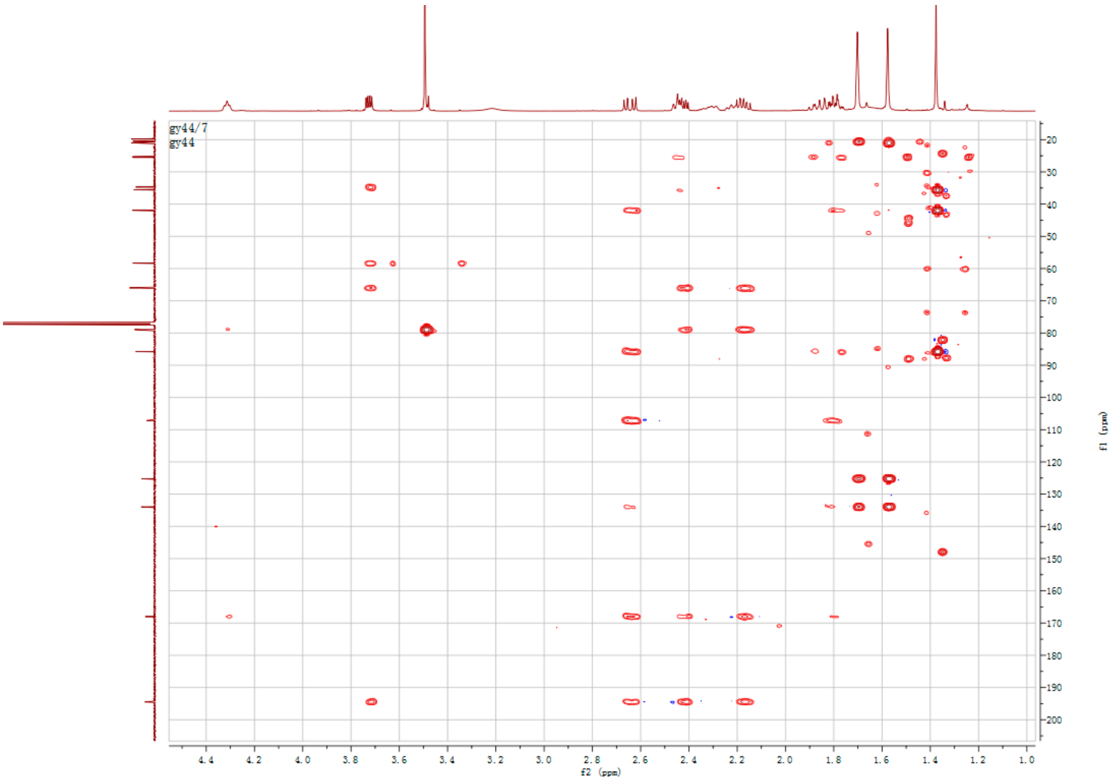

Figure S26. HMBC spectrum of guignardone S (**4**) in CDCl3.

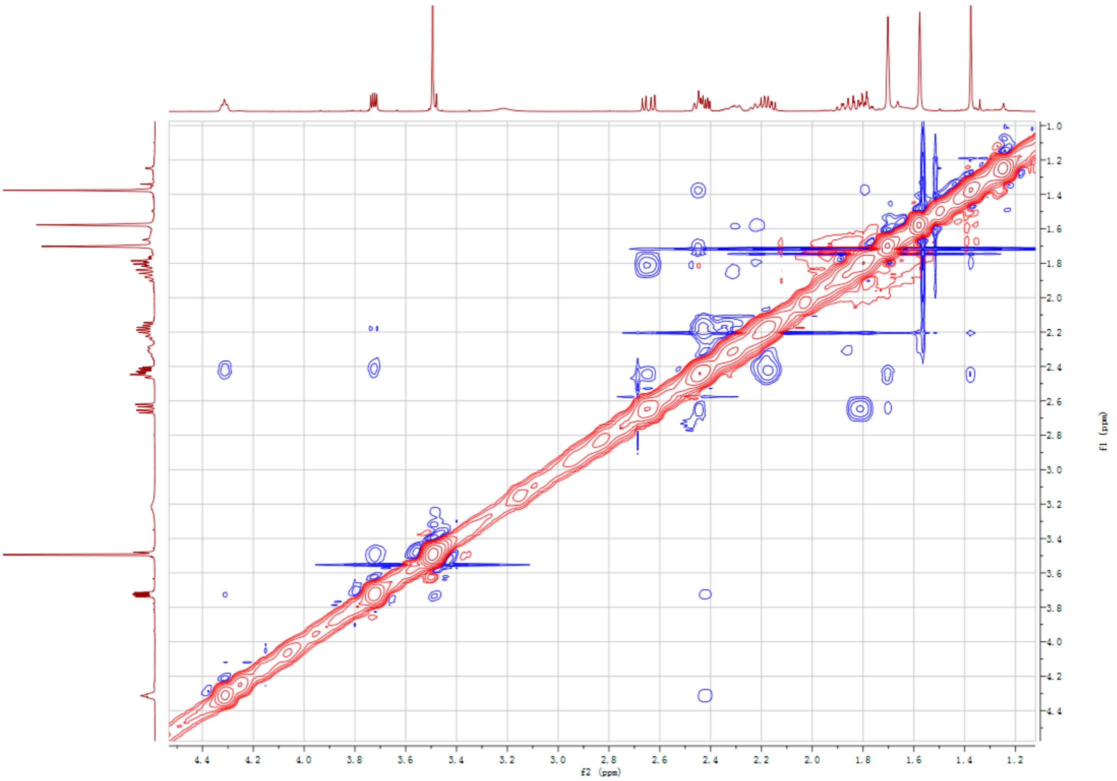

Figure S27. NOESY spectrum of guignardone S (**4**) in CDCl3.

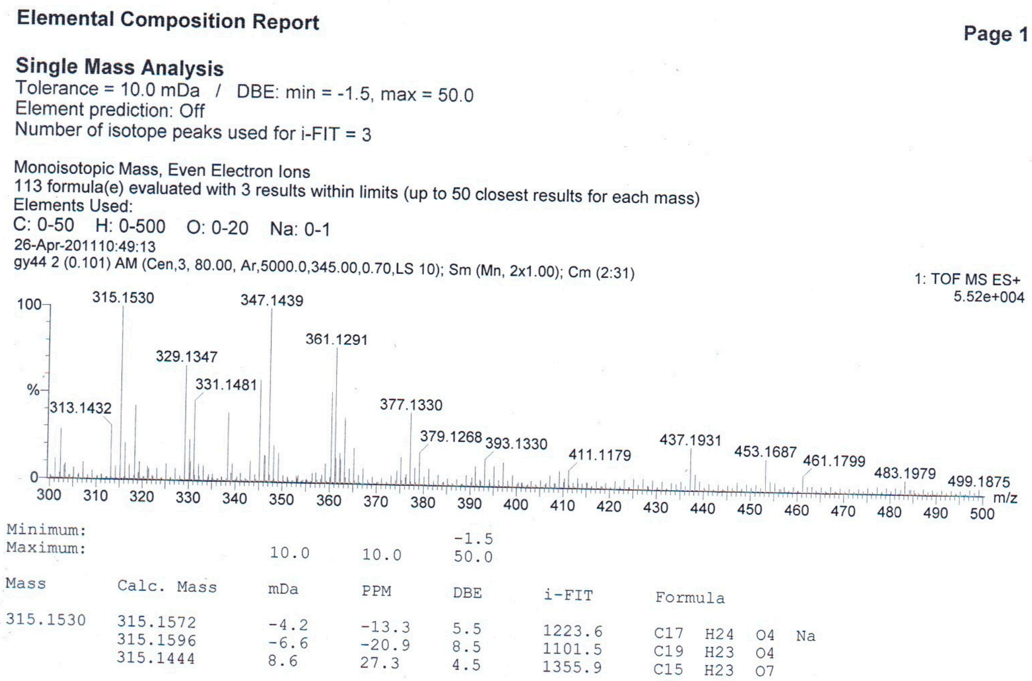

Elemental Composition Report

Page 1

Single Mass Analysis

Tolerance = 10.0 mDa / DBE: min = -1.5, max = 50.0
Element prediction: Off
Number of isotope peaks used for i-FIT = 3

Monoisotopic Mass, Even Electron Ions
113 formula(e) evaluated with 3 results within limits (up to 50 closest results for each mass)
Elements Used:
C: 0-50 H: 0-500 O: 0-20 Na: 0-1
26-Apr-201110:49:13
gy44 2 (0.101) AM (Cen,3, 80.00, Ar,5000.0,345.00,0.70,LS 10); Sm (Mn, 2x1.00); Cm (2:31)

Minimum:				-1.5		
Maximum:		10.0	10.0	50.0		
Mass	Calc. Mass	mDa	PPM	DBE	i-FIT	Formula
315.1530	315.1572	-4.2	-13.3	5.5	1223.6	C17 H24 O4 Na
	315.1596	-6.6	-20.9	8.5	1101.5	C19 H23 O4
	315.1444	8.6	27.3	4.5	1355.9	C15 H23 O7

Figure S28. HRESIMS spectrum of guignardone S (**4**).

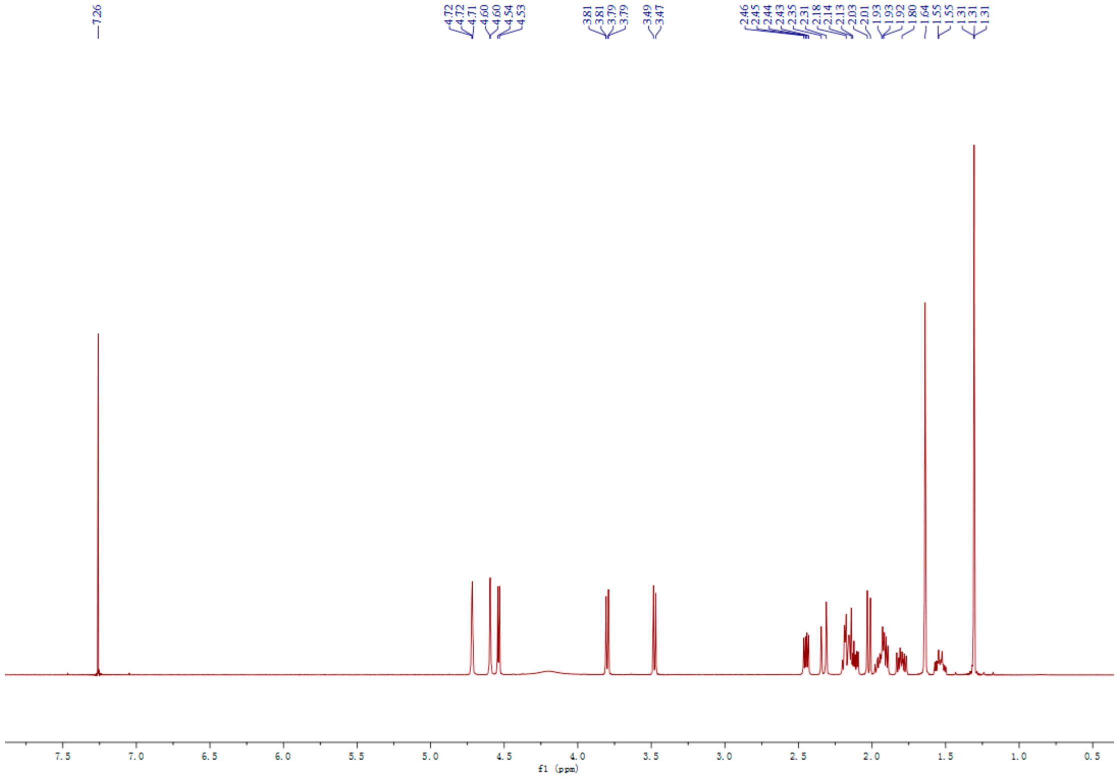

Figure S29. ^{1}H-NMR spectrum of guignardone A (**5**) in $CDCl_3$.

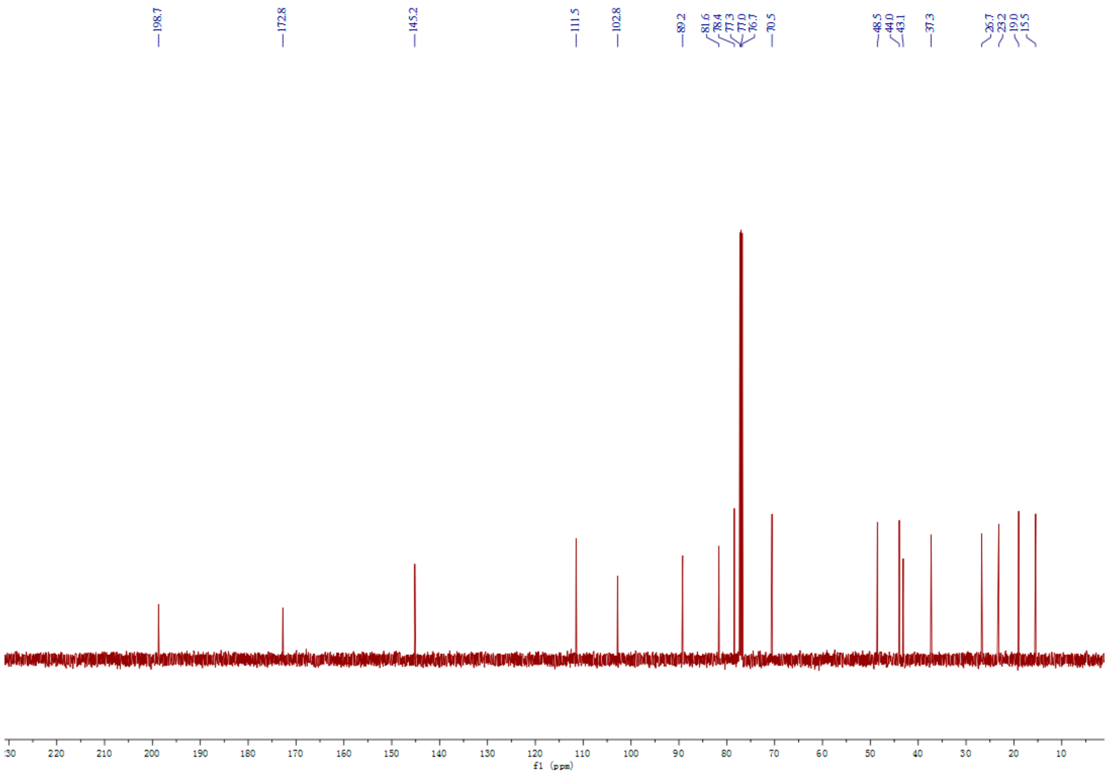

Figure S30. ^{13}C-NMR spectrum of guignardone A (**5**) in $CDCl_3$.

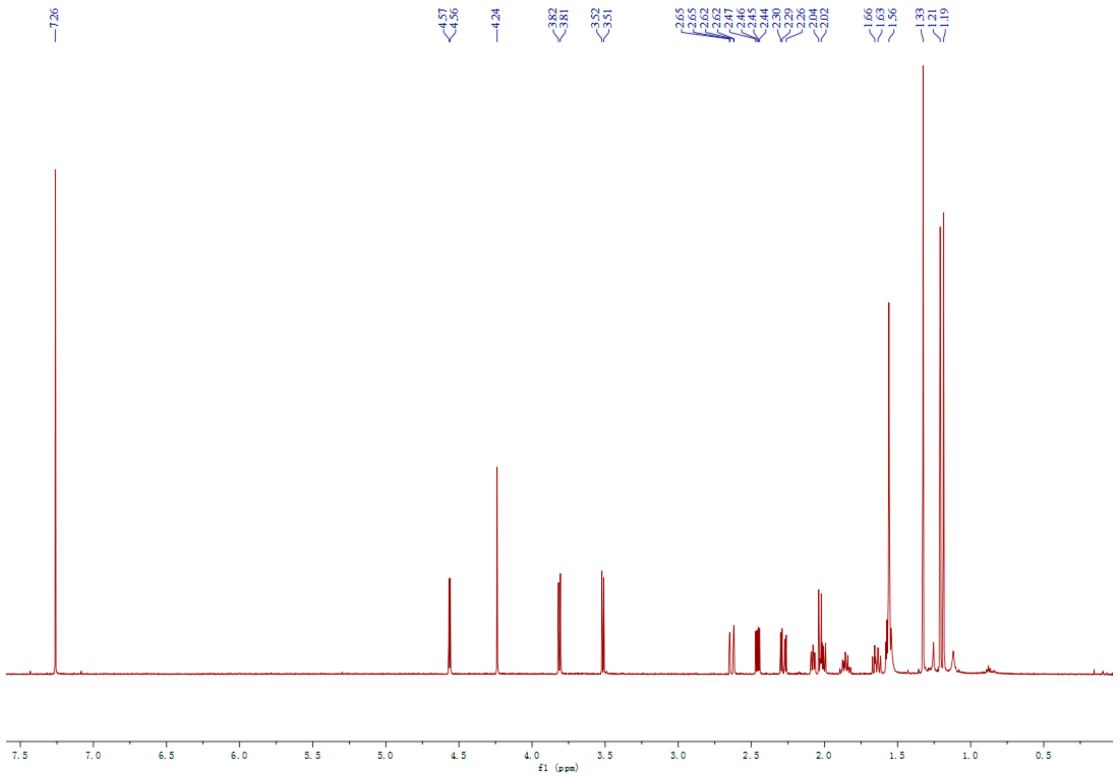

Figure S31 ^{1}H-NMR spectrum of guignardone B (**6**) in $CDCl_3$.

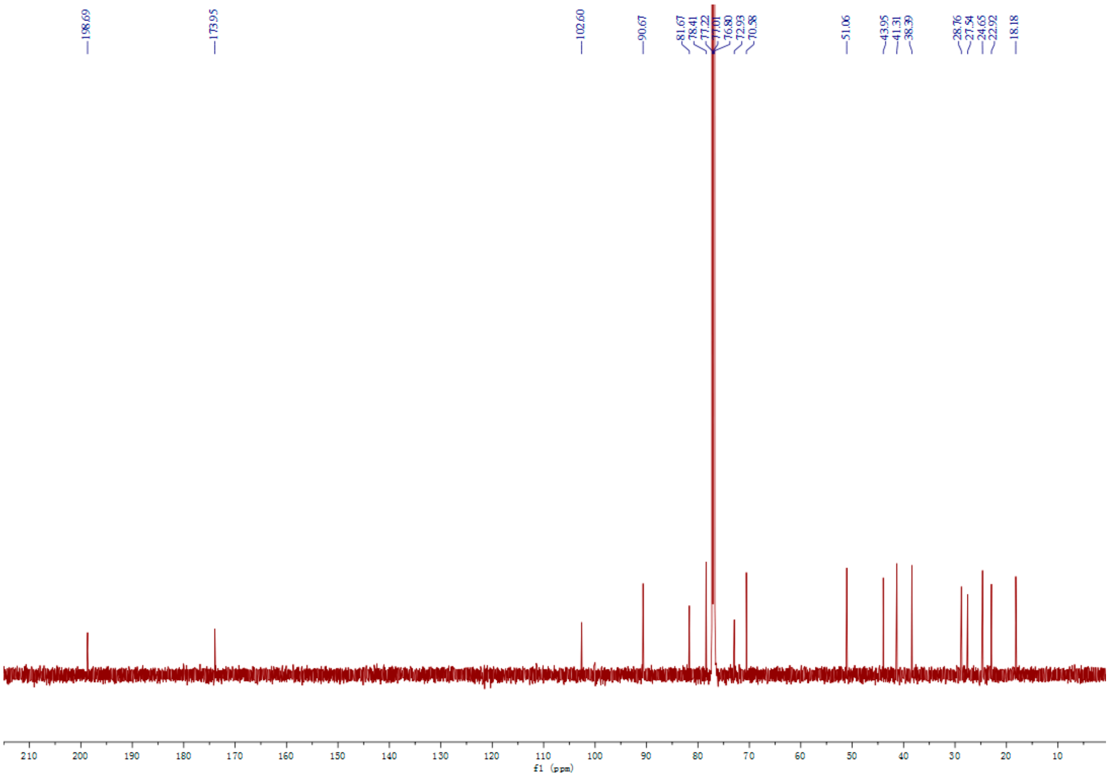

Figure S32. ^{13}C-NMR spectrum of guignardone B (**6**) in $CDCl_3$.

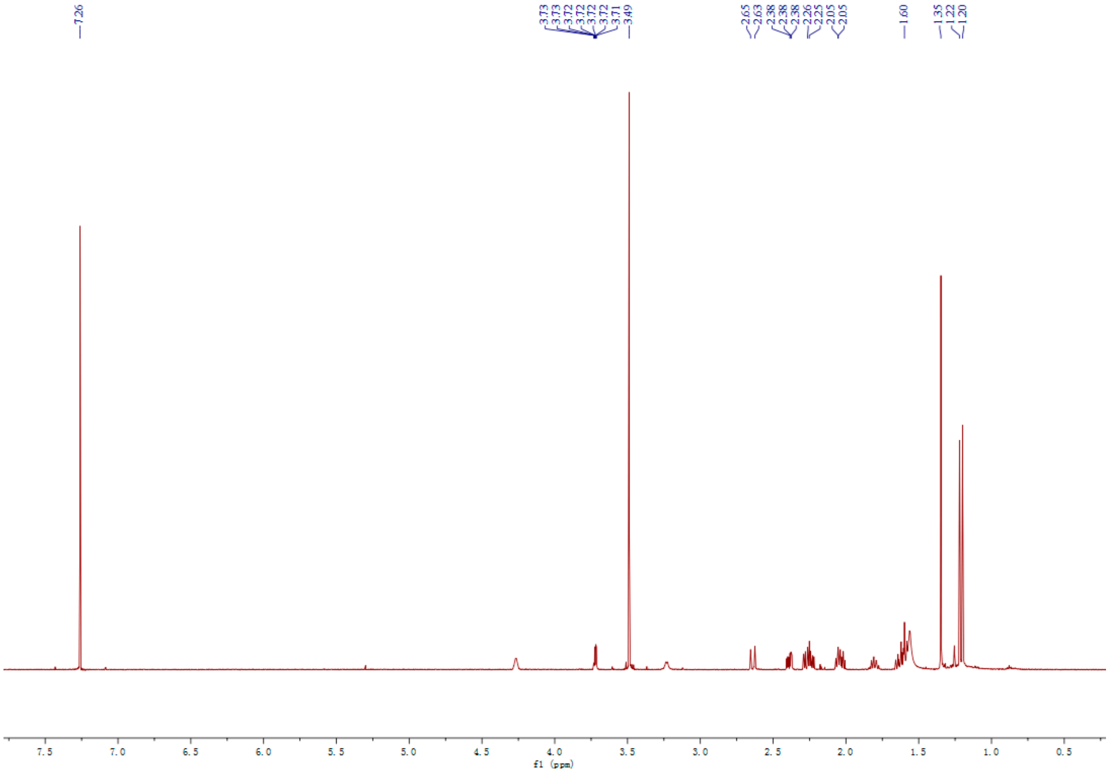

Figure S33 ^{1}H-NMR spectrum of guignardone I (**7**) in $CDCl_3$.

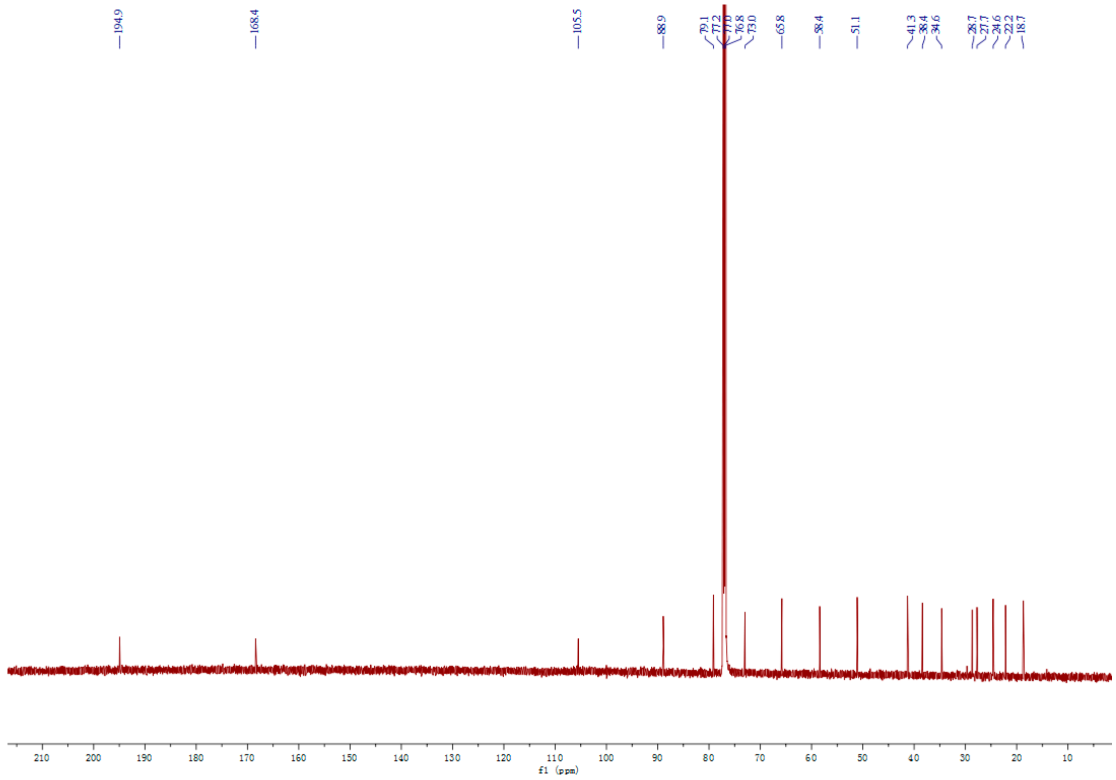

Figure S34. ^{13}C-NMR spectrum of guignardone I (**7**) in $CDCl_3$.
